# Coconut oil derived medium-chain triglycerides ameliorated memory deficits via promoting neurite outgrowth and maintaining gut homeostasis in 5×FAD mice

**DOI:** 10.3389/fnut.2025.1585640

**Published:** 2025-06-02

**Authors:** Ruiye Chen, Rui Li, Jiahui Jiang, Longjian Zhou, Shuai Zhao, Yi Zhang, Qiuyu Xia, Zhiyou Yang

**Affiliations:** College of Food Science and Technology, Guangdong Provincial Key Laboratory of Aquatic Product Processing and Safety, Guangdong Province Engineering Laboratory for Marine Biological Products, Zhanjiang Municipal Key Laboratory of Marine Drugs and Nutrition for Brain Health, Shenzhen Institute of Guangdong Ocean University, Zhanjiang, China

**Keywords:** medium-chain triglycerides, 5×FAD mice, neurite regeneration, Alzheimer’s disease, gut homeostasis

## Abstract

Alzheimer’s disease (AD) is a prevalent neurodegenerative disorder characterized by neurite atrophy, neuronal loss, and memory impairment. Medium-chain triglycerides (MCT), a type of fatty acid predominantly found in coconut oil, have been shown to improve metabolic syndrome as well as cognitive performance via ketone production in humans. Here, we investigated the protective effects of MCT on neurite atrophy and memory deficits in 5×FAD mice and elucidated the underlying mechanisms. First, virgin coconut oil (VCO), refined, bleached, and deodorized coconut oil (RBDCO), and MCT were orally administered to 6–8 months old 5×FAD mice for 9 consecutive weeks, the effects on cognition were then evaluated. MCT demonstrated superior effects compared to RBDCO and VCO in reducing Aβ levels, inhibiting hyperactivated microglia and astroglia, protecting neurons, and mitigating memory decline. Further, metagenomic analysis and RT-qPCR results revealed that MCT intervention increased the relative abundance of *Akkermansia*, reduced intestinal permeability, and elevated the concentration of short-chain fatty acids in the brain. Additionally, MCT treatment significantly protected primary cortical neurons against Aβ25-35-induced apoptosis and promoted neurite regeneration. Transcriptome and RT-qPCR data suggested that *Ucp1* and Flor1 may be potential targets through which MCT exerts its neuroprotective effects. Our findings suggest that MCT may help prevent the progression of AD by promoting neurite outgrowth and maintaining gut homeostasis in 5×FAD mice, offering a theoretical foundation for the development of dietary therapies for AD.

## 1 Introduction

Alzheimer’s disease (AD), which accounts for 60–70% of dementia cases, is a chronic neurodegenerative disorder primarily characterized by progressive cognitive decline (1). Neurofibrillary tangles, glial cell hyperactivation, senile plaques, neuroinflammation, and neuronal dysfunction are the main pathological features of AD (2, 3). The etiology of AD is intricate, but is generally attributed to several factors such as genetics, aging, and environment (4). Currently, no effective pharmacological treatments are available for AD. Dietary interventions, regarded as modifiable environmental factors, have garnered increasing research interest as an alternative approach to preventing the onset and delaying the progression of AD (5, 6). Certain foods and herbs, such as goji berries, ginseng, and yam, are regarded as neuroprotective due to their antioxidant, anti-inflammatory, and neurotrophic properties. These foods are believed to improve cognitive function by reducing oxidative stress and enhancing synaptic plasticity—two key factors involved in the pathogenesis of AD (7, 8).

In addition to medicinal foods, the ketogenic diet (KD) has garnered significant attention as a potential therapeutic strategy for AD, as it induces ketosis, which reduces neuroinflammation and enhances cognitive function. Medium-chain triglycerides (MCT), a key component of the ketogenic diet, are easily converted into ketone bodies, providing an alternative energy source for the brain. MCT have been shown to improve mitochondrial function, reduce the formation of amyloid-β plaques, and alleviate cognitive decline in both animal models and human studies (9, 10).

Coconut oil, which is rich in MCT, has been widely studied for its potential benefits in AD. The composition of coconut oil can vary depending on the processing method, leading to differences in its bioavailability and efficacy. Virgin coconut oil (VCO) retains a higher concentration of polyphenols, along with some undesirable long-chain fatty acids (LCFAs), compared to refined, bleached, and deodorized coconut oil (RBDCO). In contrast, RBDCO, typically processed at high temperatures, may have a reduced phenolic content. MCT oil derived from coconuts, primarily composed of caprylic acid and capric acid, offers greater efficiency and speed in energy conversion due to its higher concentration of readily metabolizable MCT. However, the effects of coconut-derived VCO, RBDCO, and MCT oils on memory function in 5×FAD mice remain to be fully elucidated (11, 12).

Growing evidence highlights the role of coconut oil and its derived MCT in energy metabolism, particularly in promoting ketosis. However, few studies have investigated their effects on the gut microbiome and neuronal protection. Recent studies suggest that the gut-brain axis plays a crucial role in regulating cognitive function (13). In addition, neuronal survival and neurite integrity are important for memory formation, consolidation, and retrieval. Therefore, this study aimed to compare the effects of VCO, RBDCO, and MCT on memory function, and to explore whether these oils could promote neurite outgrowth and restore gut microbiota balance in 5×FAD mice, providing a theoretical foundation for their use as a dietary intervention in AD.

## 2 Materials and methods

### 2.1 Animals and treatment

5×FAD mice (6–8 months old, female) were purchased from Jiangsu Ailingfei Biotechnology Co., Ltd. (Nanjing, China). The mice were housed in a controlled environment at 22 ± 1°C and 55 ± 10% humidity, with a 12 h light/dark cycle (starting at 7:00 AM), and had ad libitum access to food and water.

Coconut cream was purchased from Haikou Xianmeirui Industrial Co., LTD. (Haikou, China) and subjected to a freeze–thaw process (freezing at −80°C for 6 h in an HFLTP86 ultra-low temperature freezer, Heal Force Instrument Co., LTD, Shanghai, China, followed by thawing at 50°C for 2 h in a Hongze/HH-8 digital display constant temperature stirring water bath, Changzhou Hongze Experimental Technology Co., LTD, Changzhou, China) repeated 3 times. The cream was then centrifuged (HR/T20MM high speed refrigerated centrifuge, Hunan Herexi Instrument & Equipment Co., Ltd., Changsha, China) at 12,000 g for 20 min, and the procedure was repeated 3 times. The supernatant oil layer was collected and designated as virgin coconut oil (VCO). Medium-chain triglycerides (MCT) and refined, bleached, and deodorized coconut oil (RBDCO) were acquired from the Chenjia Food Technology Co., Ltd. (Shanghai, China). 5×FAD mice and age-matched wild-type (WT) littermates were randomly assigned to the following groups: control (Cont, WT mice, *n* = 7), vehicle (Veh, 5×FAD mice, *n* = 7), VCO (5×FAD mice, *n* = 6), RBDCO (5×FAD mice, *n* = 7), and MCT (5×FAD mice, *n* = 6). Mice were orally gavaged with saline (Cont and Veh), VCO, RBDCO, or MCT (8.2 g/kg/day) for 9 consecutive weeks. The dosages were determined based on the literature (14).

### 2.2 Behavioral test

The novel object recognition test (ORT) and the object location test (OLT) are used to assess recognition and spatial memory, as previously described (15). The Morris Water Maze (MWM) test was employed to evaluate spatial learning and memory in mice. In this test, a circular pool (diameter: 150 cm) is divided into four quadrants, with a transparent platform placed in one of them. The pool is filled with water containing food-grade titanium dioxide to obscure the platform’s location. Each mouse is sequentially placed into the pool from one of the quadrants, with each training session lasting 60 s. If the mouse fails to locate the platform within the 60-s time limit, it is guided to the platform and allowed an additional 20 s to reinforce the learning process. After a five-day training period, the platform is removed, and the mouse is placed in the diagonal quadrant for 60 s of free exploration. A probe trial is then conducted for each mouse, and the number of entries into the target quadrant is recorded using the Supermaze analysis system (Supermaze Software, Xinruan Information Technology Co., Ltd., Shanghai, China).

### 2.3 Evaluation of pharmacological indicators

#### 2.3.1 ELISA analysis

Following the behavioral tests, the mice were deeply anaesthetized with a mixture of methamphetamine hydrochloride (23 mg/kg), zolazepam (15 mg/kg), and salbutamol hydrochloride (15 mg/kg) via intraperitoneal injection. Approximately 1.0 mL of blood was collected from the abdominal aorta and centrifuged at 2,500 g for 15 min to obtain serum. The ELISA kits for detecting IL-1β, IL-4, IL-6, and IL-10 were purchased from Zeyu Biotech Co., Ltd. (Jiangsu, China). The serum concentrations of these cytokines were measured according to the manufacturer’s instructions.

M-PER™ Mammalian Protein Extraction Reagent (M-PER, Thermo Scientific, #78501) was used to lyse brain tissues, while a protease inhibitor cocktail (1×, Thermo Scientific, #87786) was used to inhibit protein degradation. The lysate was incubated for 30 min on ice, and then centrifuged (12,000 g, 4°C) for 10 min. The supernatants were obtained and total proteins were quantified using Pierce™ 660 nm Protein Assay Reagent (Thermo Scientific, #1861426). IL-1β, IL-4, IL-6, and IL-10 were determined by ELISA kits (Meimian, Yancheng, China).

#### 2.3.2 Immunohistochemistry

The left hemisphere was fixed in 4% PFA for 48 h, followed by immersion in 10, 20, and 30% sucrose to facilitate gradient dehydration. The tissues were embedded in OCT embedding medium (Sakura Tissue-Tek® O. C. T.) and sectioned into15 μm slices using a cryostat (Kedee, Jinhua, China). The brain sections were subsequently fixed with 4% PFA at room temperature for 90 min, followed by three 5-min washes with PBS containing 0.2% Triton X-100. Primary antibodies against Aβ (Invitrogen, 1:500, 700,254), Iba1 (Wako, 1:500, 019–19,741), GFAP (Invitrogen, 1:200, MA5-12023), and NeuN (Abcam, 1:500, ab177487) were applied and incubated overnight at 4°C. Afterward, secondary antibodies, Alexa Fluor 594 (Abcam, 1:500, ab150116) and Alexa Fluor 488 (Abcam, 1:500, ab150081), were applied and co-stained with 4′,6-diamidino-2-phenylindole (DAPI, 1 μg/mL, MCE, USA) for 2 h at room temperature in the dark. Fluorescence images were captured using a fluorescent microscope (ECHO Revolve, CA, USA) at a magnification of 477 μm × 636 μm. The captured images were then analyzed using ImageJ software (NIH, Bethesda, Maryland, USA).

#### 2.3.3 RT-qPCR analysis

Total RNA was extracted from the right hemisphere or colon using the AG RNAex Pro Reagent (Accurate Biology, Hunan, China). RNA quality and concentration were determined using the KLP05-6 Nucleic Acid Quantifier (DeNovix Inc., USA). cDNA synthesis was performed with the HiScript III Q Select RT SuperMix for qPCR (+gDNA wiper) kit (Vazyme). Polymerase chain reaction (PCR) amplification was carried out using the ChamQ Universal SYBR qPCR Master Mix Kit (Q711, Vazyme) on a CFX96Touch™ Real-Time PCR System (Bio-Rad, USA). The initial activation step was performed at 95°C for 30 s, followed by 40 cycles of amplification (95°C for 5 s and 60°C for 30 s). Data were analyzed using the 2-ΔΔCT method, with glyceraldehyde-3-phosphate dehydrogenase (*GAPDH*) as the internal reference gene. The primers were designed and synthesized by Sangon Biotechnology (Shanghai, China), and the sequences are listed in Table 1.

**Table 1:** Primer sequences for qPCR.

Gene name	Forward primer (5′ to 3′)	Reverse primer (5′ to 3′)
*GAPDH*	CAGCAAGGACACTGAGCAAG	GGTCTGGGATGGAAATTGTG
*Occludin*	TTGACTGGGCTGAACACTCC	ACATCACAGCTCACACCAGG
*ZO-1*	AAACAGCCCTACCAACCTCG	TTCGAGGCAGCTGCTCATAG
*Bcl-2*	GCTACCGTCGTGACTTCGC	CCCAGCCTCCGTTATCC
*Bax*	TCATCCAGGATCGAGCAGG	GCAAAGTAGAAGAGGGCAACC
*Ucp1*	TTGGGCTTCTATGCTGGGAG	GTGAATGCTATGCTCTTCTGTCT
*Gpx2*	CAGGGCTGTGCTGATTGA	CAAGGGAAGCCGAGAACTA
*Folr1*	AGTTGTTGCTCCTGGTGATG	TTATGTGCTTCCTGGCTTG
*Myod1*	ACATGGGAGCCCTCCTGAAA	CCTTGGGTAGCCGCTGGTT

#### 2.3.4 Short-chain fatty acid measurement

Fifty milligrams of brain tissue were homogenized with 50 μL of pre-cooled 15% phosphoric acid, and then vortexed after adding 50 μL of the internal standard DL-2-methylbutyric acid (1 mg/mL). Next, 900 μL of ethyl acetate was added, and the sample was vortexed for 1 min. The mixture was then centrifuged at 4°C and 12,000 g for 10 min. The supernatant was treated with anhydrous sodium sulfate and vortexed to remove water. The sample was subsequently filtered through a 0.45 μm membrane filter and analyzed using a triple-quadrupole gas chromatograph-mass spectrometer (GCMS-TQ8040NX, SHIMADZU, Kyoto, Japan).

The chromatographic conditions are as follows: an InertCap Pure-WAX capillary column (30 m × 0.25 mm × 0.25 μm) was used. Helium was used as the carrier gas with a flow rate of 1.0 mL/min. A split injection mode was employed with a split ratio of 40:1, and the injection volume was 1 μL. The injector temperature was set to 250°C. The temperature program was as follows: the initial temperature was 60°C, held for 5 min, then increased at a rate of 10°C/min to 110°C, followed by an increase at 35°C/min to 250°C, and held for 1 min.

The mass spectrometric conditions were as follows: electron ionization (EI) was used with selected ion monitoring (SIM) mode for scanning. The electron energy was set to 70 eV. The ion source temperature was set to 230°C, the transfer line temperature to 250°C, and the quadrupole temperature to 150°C.

### 2.4 16S rRNA sequencing

Fresh fecal samples were collected from the mouse cecum and placed into sterile EP tubes. DNA was extracted using the HiPure Stool DNA Extraction Kit (Magen, Guangzhou, China). The quality of the extracted DNA was assessed by 2% agarose gel electrophoresis. The bacterial 16S rRNA gene was amplified using primers 341F (CCTACGGGNGGCWGCAG) and 806R (GGACTACHVGGGTATCTAAT) for the V3 and V4 hypervariable site. The PCR products were purified using AMPure XP beads (Beckman, CA, USA) and quantified with a Qubit 3.0 fluorometer (Life Technologies, CA, USA), then sequenced on an Illumina NovaSeq6000 platform (Genedenovo Biotech, Guangzhou, China). The raw sequencing reads were de-replicated, and low-quality data were filtered out using FASTP software. Metagenomic data were analyzed using the Statistical Analysis of Metagenomic Profiles (STAMP) software package (v2.1.3).

### 2.5 Chemical analysis of VCO, RBDCO, and MCT

#### 2.5.1 Fatty acid composition analysis

The oils were converted to fatty acid methyl esters (FAMEs) following the Chinese National Standard GB 5009.168–2016 and identified using pure standards. The fatty acid composition was analyzed using an Agilent 7,890–5,975 gas chromatography–mass spectrometry (GC–MS) system (Agilent Tech., Wilmington, USA). Specifically, the FAMEs of the three samples were prepared by transesterification. A HP-5MS column (60 m × 0.25 mm i.d., 0.25 μm film thickness) was used. The operating conditions were as follows: the injector temperature was set to 280°C, and the injection volume was 1.0 μL. A temperature program was applied, starting with an initial column temperature of 120°C, held for 1 min. The temperature was then increased to 170°C at a rate of 6°C/min, further raised to 215°C at 2.5°C/min, and held for 12 min. Next, the temperature was increased to 230°C at 4°C/min and held for 10 min, and finally raised to 280°C at 10°C/min, holding for 15 min. The helium carrier gas flow rate was 1.5 mL/min, with a split ratio of 20:1. The mass spectrometer was operated at an ionization voltage of 70 eV and an ion source temperature of 200°C. The quadrupole temperature was set to 150°C, and the scanning range was m/z 40–550. Fatty acids were identified using the National Institute of Standards and Technology (NIST) 11 mass spectrum database, based on fatty acid standard spectra and retention time. The results were expressed as the percentage of individual fatty acids.

#### 2.5.2 Phenolic acids analysis

Two grams of each oil were placed into separate 10 mL centrifuge tubes, and 5 mL of a 70% methanol solution was added to each tube. The polyphenols were extracted after mixing thoroughly and standing for 3 h. Ultrasonic treatment was then applied for 30 min, followed by centrifugation at 8000 g for 10 min using a H/T16MM desktop high-speed centrifuge (Hunan Herexi Instrument & Equipment, Changsha, China). The supernatant, containing polyphenolic acids, was collected and transferred to a vial. The polyphenolic acid components were subsequently analyzed by liquid chromatography–tandem mass spectrometry (LC–MS/MS).

LC–MS/MS analysis was performed using an Agilent 1,100 high-performance liquid chromatography system coupled to a Triple Quadrupole API 4000 mass spectrometer (Agilent Technologies, Inc., Wilmington, USA). A 10 μL sample was injected into an Agilent Poroshell 120 EC-C18 column (50 mm × 3 mm i.d., 2.7 μm) maintained at 35°C. The ternary mobile phase comprised (A) methanol with 0.5% formic acid/H_2_O and (B) acetonitrile, and was set at a flow velocity of 0.6 mL/min in gradient elution. The ratios of A and B were initially set to 95%:5%, and held for 1 min; gradually changed to 75%:25% within 7 min, and changed to 40%:60% within 4 min; phase B was increased to 100% within 1 min and held for 3 min; then immediately changed back to 95%:5% within 0.1 min and maintained for 4 min. Electrospray ionization (ESI) was used with spray voltages of 5.5 kV (ESI^+^) and 4.5 kV (ESI^−^). The vaporization temperature was set to 500°C, and the nitrogen sweep gas flow rate was 1,000 L/h.

#### 2.5.3 Tocopherols analysis

Samples were weighed and transferred into a 25 mL volumetric flask. To each sample, 0.1 g of butylated hydroxytoluene (BHT) was added, and the mobile phase solution, composed of 3.75% tetrahydrofuran and 96.25% n-heptane (v/v), was used to dissolve the samples by oscillation. Prior to analysis, the samples were filtered using a 0.45 μm Millipore filter.

Tocopherols were analyzed using an Agilent 1,100 HPLC (Agilent Technologies, Inc., Wilmington, USA), equipped with a variable wavelength detection (VWD) detector (Agilent Technologies, Inc., Palo Alto, USA). Separation was carried out on a Welch Ultimate XB-Diol column (4.6 mm × 250 mm, 5 μm, Yuexu Technology Co., Ltd., Shanghai, China), maintained at 30°C. The mobile phase, composed of 3.75% tetrahydrofuran and 96.25% n-heptane (v/v), was delivered at a flow rate of 1.0 mL/min. The VWD detector was set to 292 nm.

### 2.6 Primary neuron culture and bioactive analysis

#### 2.6.1 Primary cortical neuron culture

The primary cortical neurons were isolated from ICR mouse embryos (14 days gestation) as previously described (16). Briefly, the dura mater was carefully removed from the cerebral cortex, which was then subjected to enzymatic digestion using 0.5% trypsin–EDTA (Gibco, 25,300,054) to isolate the neurons. The neurons were seeded in Neurobasal medium (Gibco, 21,103–049) supplemented with 0.6% D-glucose, 2 mM L-glutamine, and 2% B27. Neurons were cultured at a density of 2.1 × 10^4^ cells/cm^2^ in 96-well plates for cell viability assays, or in 48-well plates for neurite outgrowth assays.

#### 2.6.2 Neuronal viability assay

After 3 days of neuronal culture, neurons were treated with 10 μM Aβ25-35 (Sigma, A4559) for 30 min, followed by the addition of MCT or RBDCO (1, 10, 100, and 1,000 μg/mL) for 48 h. Subsequently, 10 μL of Cell Counting Kit (CCK8, APEXBIO, K1018) reagent was added to each well and incubated for 3 h, according to the manufacturer’s instructions. The fluorescence absorbance was measured at 450 nm using a 96-well ELISA reader (Biotek, VT, USA). Aβ25-35 was dissolved in deionized water to a final concentration of 5 mM and incubated at 37°C for 4 days to facilitate aggregation.

#### 2.6.3 Neurite outgrowth measurement

After 3 days of neuronal culture, neurons were treated with 10 μM Aβ25-35 for an additional 3 days. Fresh medium containing MCT (10, 100, and 1,000 μg/mL) was then applied for 4 days. Following treatment, neurons were fixed with 4% PFA at 25°C for 1 h and subsequently underwent immunostaining for pNF-H (1:500, Covance, SMI-35R, CA, USA) and MAP2 (1:2000, Abcam, ab32454). The secondary antibodies employed were Alexa Fluor 594 and Alexa Fluor 488, and nuclear counterstaining was performed with DAPI. Images were captured using a fluorescence microscope system (ECHO, San Diego, CA, USA). Ten images were collected for each group and analyzed using ImageJ (NIH) software with the neuron tracking plugin. The mean length of pNF-H or MAP2-positive axons and dendrites was determined for each neuron.

#### 2.6.4 Transcriptome analysis

Primary cortical neurons were cultured for 3 days, after which 10 μM Aβ25-35 was added for 30 min. The neurons were then incubated with 1,000 μg/mL MCT for 3 h. Total RNA was extracted using the TRIzol kit, and its purity was evaluated with a NanoPhotometer® spectrophotometer (IMPLEN, CA, USA). RNA concentration and integrity were determined using the Qubit® 3.0 Fluorometer (Life Technologies, CA, USA) and the Agilent 2,100 RNA Nano 6,000 Assay Kit (Agilent Technologies, CA, USA), respectively. mRNA was enriched using magnetic beads with Oligo (dT), and the synthesized cDNA was subjected to PCR amplification and sequencing on the Illumina PE150 platform. Differential gene expression was analyzed using HISAT2 software (*p* < 0.05).

### 2.7 Western blot analysis

Brain and colon tissues of mice were homogenized with M-PER (Thermo Scientific) containing 1 × protease inhibitor mixture (Thermo Scientific). Tissues were homogenized and incubated on ice for 30 min. The lysates were centrifuged (12,000 g, 4°C) for 10 min. The supernatant was collected and the total proteins were determined using the Pierce™ 660 nm Protein Assay Kit. Three samples of each group were loaded into SDS-PAGE and then transferred to a PVDF membrane. The PVDF membrane was blocked with 5% skim milk for 1 h at room temperature. The membranes were incubated overnight at 4°C with primary antibodies MAP2 (Abcam, #ab32454), PSD95 (Thermo Scientific, #51–6,900), ZO-1 (Affinity, #DF7504), Occludin (Affinity, #AF5145), and β-actin (Santa Cruz, #sc47778). The membranes were washed with TBST for 6 times (3 times for 5 min and 3 times for 15 min), and were incubated with secondary antibodies (HRP-conjugated anti-mouse or anti-rabbit IgG) for 1 h at room temperature. After washing with TBST for 6 times (3 times for 5 min and 3 times for 15 min), the proteins on membrane were immunostained using high-sensitivity ECL chemiluminescent reagent (F03, Willget Biotechnology, Hangzhou, China). The grayscale value of each band was analyzed using ImageJ.

### 2.8 Statistical analysis

Data were analyzed using GraphPad Prism 9 software (GraphPad Software, California, USA). All values are presented as the mean ± SEM, and statistical analysis was performed using one-way or two-way ANOVA, followed by Dunnett’s test. A *p*-value of less than 0.05 was considered statistically significant.

## 3 Results

### 3.1 MCT ameliorated recognition and spatial memory deficits in 5×FAD mice

**Figure 1:**
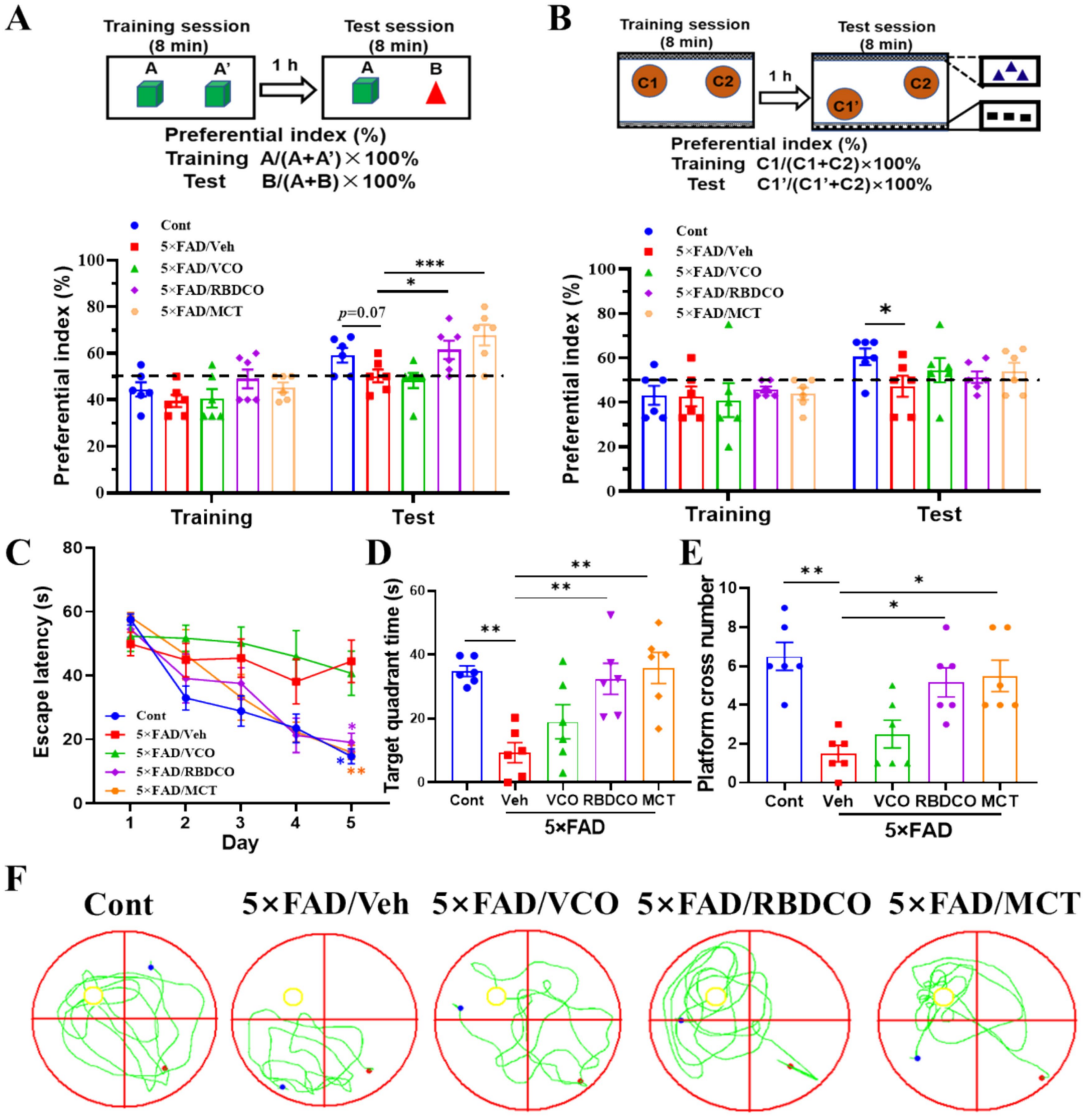
MCT improves memory deficits in 5×FAD mice. **(A)** Object recognition memory test. **(B)** Object location test. **(C)** Escape latency during the first 5 days of training. **(D)** Time spent in the target quadrant during the MWM test. **(E)** Platform crossings during the MWM test. **(F)** Representative trajectory in the MWM test (red dot: starting position, blue dot: ending position). ^*^p < 0.05, ^**^p < 0.01, ^***^p < 0.001 compared to the Veh group. Two-way ANOVA followed by Dunnett’s test (mean ± SEM, *n* = 6).

To assess the effects of VCO, RBDCO, and MCT on memory function in 5×FAD mice, object recognition test (ORT) and object location tests (OLT) were conducted on days 34 and 36, with a 1-h interval between the training and test sessions (Supplementary Figure 1). Compared to the Cont group, the Veh group showed a lower preferential index for novel objects or newly placed objects. In contrast, RBDCO and MCT treatments significantly increased the preferential index for novel objects in the ORT test, with MCT demonstrating superior effects over RBDCO (Figure 1A). Surprisingly, VCO did not improve recognition memory, and VCO, RBDCO, and MCT treatments did not improve the spatial memory in the OLT (Figure 1B).

The Morris water maze test was performed to ascertain whether MCT improved learning and memory abilities in 5×FAD mice. During the training phase, vehicle-treated 5×FAD exhibited a significant increase in escape latency compared to Cont mice, particularly on the fifth day. In contrast, RBDCO and MCT treatments significantly reduced escape latency (Figure 1C). On the sixth day, following platform removal, mice treated with RBDCO and MCT, and wild-type mice spent more time in the target quadrant and crossed the platform location more frequently compared to vehicle-treated 5×FAD mice (Figures 1D–F). Collectively, these results suggest that RBDCO and MCT have the potential to alleviate cognitive deficits in 5×FAD mice. Notably, VCO did not improve memory function, prompting further analysis focusing on the potential mechanisms of RBDCO and MCT in the treatment of AD.

### 3.2 MCT alleviated Aβ plaque accumulation and neuroinflammation in 5×FAD mice

**Figure 2:**
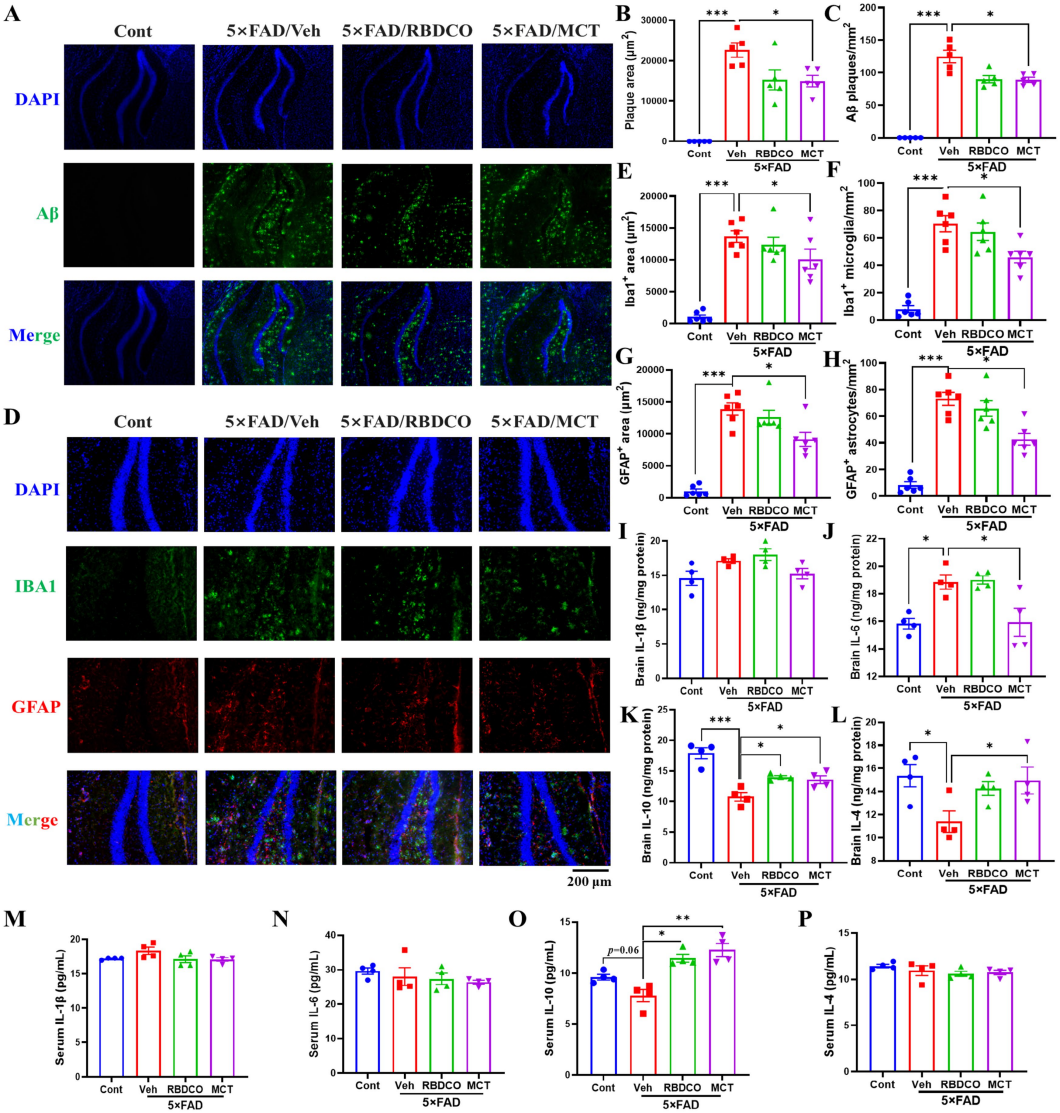
MCT alleviates Aβ plaque accumulation and neuroinflammation in 5×FAD mice. **(A)** Representative images of Aβ immunofluorescence in the hippocampal DG region of mice (scale bar: 200 μm). **(B)** The total area of plaques per μm^2^ in the DG region of the hippocampus. **(C)** The number of Aβ plaques per mm^2^ in the DG region of the hippocampus. **(D)** Representative images of Iba1 and GFAP immunofluorescence in the hippocampal DG region of mice (scale bar: 200 μm). **(E)** The area of Iba1-positive cells (μm^2^) in the DG region of the hippocampus. **(F)** The number of Iba1-positive microglia per mm^2^ in the DG region of the hippocampus. **(G)** The area of GFAP-positive cells (μm^2^) in the DG region of the hippocampus. **(H)** The number of GFAP-positive astrocytes per mm^2^ in the DG region of the hippocampus. **(I–L)** ELISA analysis of the brain levels of IL-1β, IL-4, IL-6, and IL-10. **(M–P)** ELISA analysis of the serum levels of IL-1β, IL-4, IL-6, and IL-10. ^*^p < 0.05, ^**^p < 0.01, ^***^p < 0.001 compared to the Veh group. One-way ANOVA followed by Dunnett’s test (mean ± SEM, *n* = 4–5).

Amyloid-β (Aβ), a hallmark pathological feature of AD, accumulates early in the disease process and contributes to neuronal death (17, 18). To investigate the effects of RBDCO and MCT on Aβ clearance, we employed immunofluorescence staining to detect Aβ plaques in the hippocampus of mice. Compared to Cont mice, vehicle-treated 5×FAD mice exhibited a significant increase in both the plaque area and the number of plaques in the hippocampus (Figures 2A–C). In contrast, MCT intervention markedly reduced Aβ deposition in the hippocampal region, while RBDCO treatment showed a decreasing trend in Aβ plaques. These findings suggest that MCT may help mitigate the accumulation of Aβ plaques.

Under normal physiological conditions, microglia and astrocytes exist in a resting or quiescent state. However, toxins can trigger glial proliferation and hyperactivation in the pathogenesis of AD. Previous studies have shown that glial hyperactivation plays a critical role in accelerating Aβ deposition, neuroinflammation, and oxidative stress (19). Therefore, we employed immunofluorescence staining to detect the reactive glial markers IBA1 (for microglia) and GFAP (for astrocytes) in the hippocampus. Compared to the Cont group, the vehicle-treated 5×FAD mice exhibited a notable increase in the number of IBA1- and GFAP-positive cells and their expression in the hippocampus (Figures 2D–H). In contrast, MCT treatment significantly reduced the expression of IBA1 and GFAP, as well as the hyperactivation of microglia and astrocytes. It has been demonstrated that peripheral inflammation can disrupt the blood–brain barrier (BBB) through various pathways, leading to neuroinflammation (20). In addition, cytokine levels were measured using ELISA in brain tissues. Compared to Cont mice, the vehicle 5×FAD mice exhibited elevated levels of IL-1β and IL-6, and reduced levels of IL-4 and IL-10. MCT treatment upregulated IL-4 and IL-10 expression, and downregulated IL-1β and IL-6 levels (Figures 2I–L). Furthermore, the levels of cytokines in serum were also evaluated, the vehicle 5×FAD mice exhibited an increased trend in IL-1β and a decreased level of IL-10 compared to Cont mice. Treatment with RBDCO and MCT attenuated the hyperactivated immune response, as evidenced by the upregulation of IL-10 and a reduced trend in IL-β levels, compared to vehicle-treated 5×FAD mice (Figures 2M,O). No statistically significant differences were observed in IL-6 and IL-4 levels between the groups (Figures 2N,P). These results suggest that MCT inhibits both peripheral and neuronal inflammatory responses.

### 3.3 MCT alleviated neuronal apoptosis in 5×FAD mice

**Figure 3:**
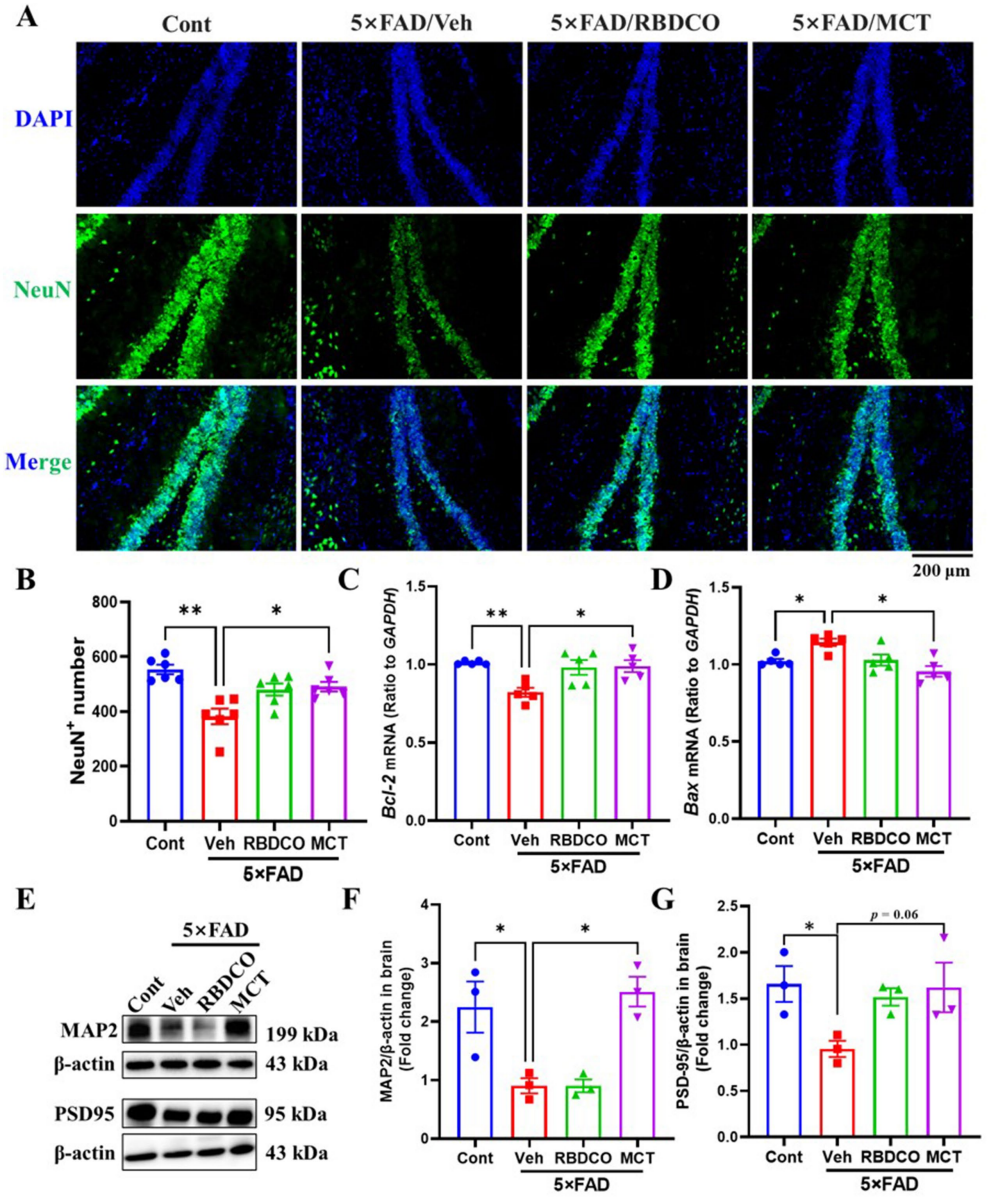
MCT alleviates neuronal damage in 5×FAD mice. **(A)** Representative NeuN immunostaining images of the hippocampal DG in mice (scale bar: 200 μm). **(B)** The number of NeuN-positive cells in the hippocampal DG. **(C,D)** mRNA expression levels of *Bcl-2* and *Bax* in mouse brain tissue. **(E–G)** Immunoblot analysis of MAP2 and PSD95 in the brain. ^*^p < 0.05, ^**^p < 0.01, ^***^p < 0.001 compared to the Veh group. One-way ANOVA followed by Dunnett’s test (mean ± SEM, *n* = 3–5).

Hippocampal neuronal death impairs synaptic function and disrupts neural networks, leading to cognitive failure in AD (21). To evaluate the neuroprotective effects of MCT in 5×FAD transgenic mice, we performed immunohistochemical staining using the NeuN antibody. Compared to the Cont group, the vehicle-treated 5×FAD mice exhibited a significant reduction in the number of NeuN^+^ neurons in the hippocampal region (Figures 3A,B). In contrast, MCT intervention dramatically increased the number of hippocampal neurons, while RBDCO treatment showed an increasing trend in neuron numbers compared to the vehicle-treated 5×FAD mice (Figures 3A,B).

*Bcl-2* and *Bax* are cytoplasmic proteins with opposing effects on neuronal apoptosis. *Bcl-2* has been shown to inhibit cell death, whereas *Bax* promotes apoptosis (22). The messenger RNA (mRNA) expression levels of *Bcl-2* and *Bax* were quantified in the mouse hippocampus using Real-Time quantitative PCR (RT-qPCR). Compared to the Cont group, vehicle-treated 5×FAD mice exhibited decreased *Bcl-2* mRNA levels and increased *Bax* mRNA expression (Figures 3C,D). In contrast, MCT demonstrated superior effects to RBDCO in upregulating *Bcl-2* mRNA expression and reducing *Bax* mRNA levels, compared to vehicle-treated 5×FAD mice.

Microtubule-associated protein 2 (MAP2), a dendritic marker predominantly expressed in mature neurons, plays crucial roles in maintaining neuronal cytoarchitecture and regulating axonal elongation, synaptogenesis, and neuroplasticity (23). Postsynaptic density protein 95 (PSD-95), a scaffolding protein enriched in the postsynaptic membrane compartment, serves as a critical regulator of synaptic maturation and functional stabilization (24). Our western blot analysis demonstrated that MCT intervention significantly enhanced MAP2 expression levels (Figures 3E,F), suggesting potential reinforcement of neuronal cytoskeletal integrity that may facilitate axonal regrowth and morphological restoration. While the increase in PSD-95 expression did not attain statistical significance (*p* = 0.06) (Figures 3E,G), the observed upward trend implies partial synaptic reorganization might occur following MCT treatment. These findings collectively indicate that MCT-mediated neurostructural recovery, through cytoskeletal stabilization and synaptic remodeling, could create a permissive microenvironment conducive to axonal regeneration.

### 3.4 MCT maintained gut homeostasis in 5×FAD mice

**Figure 4:**
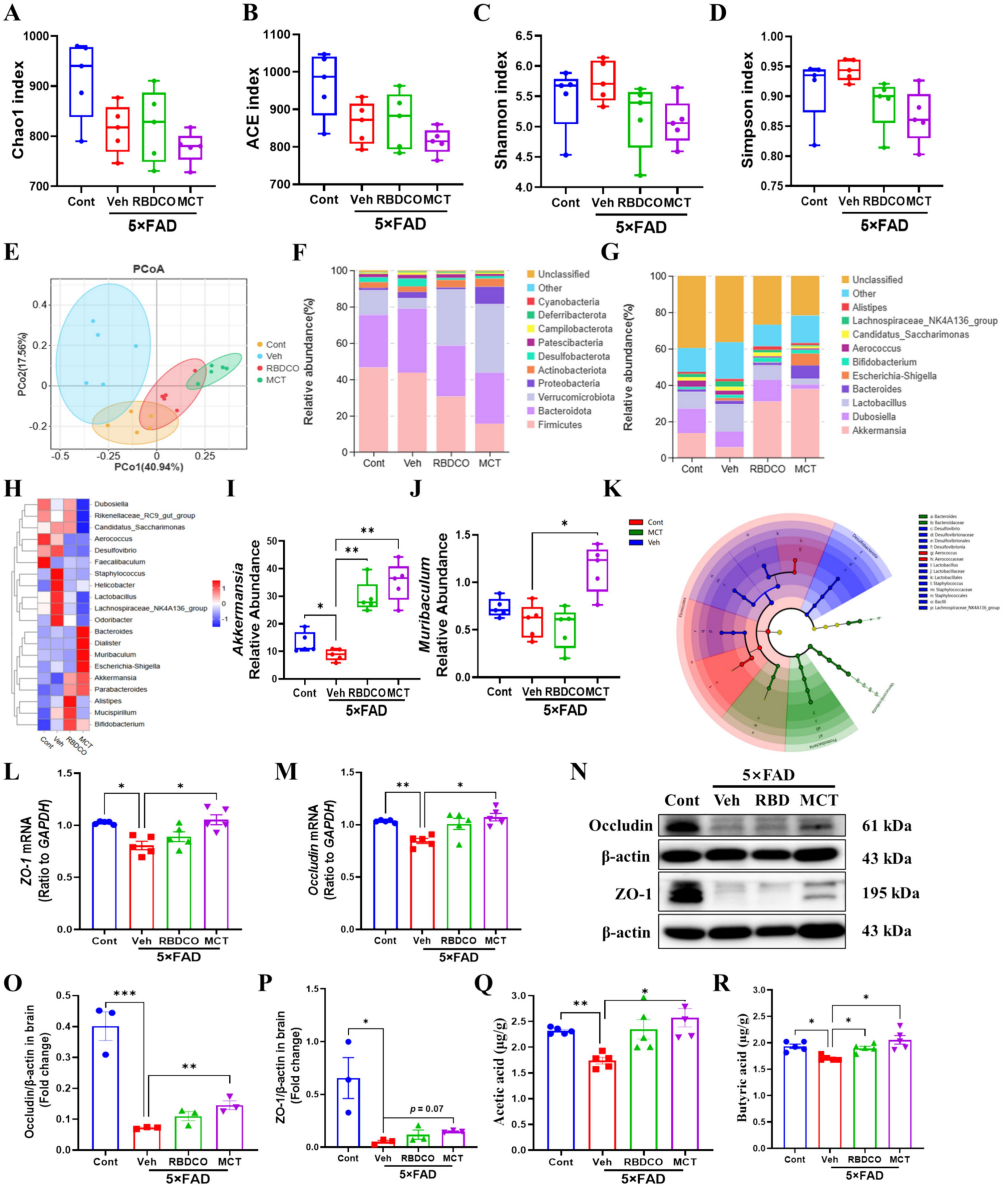
MCT alters the gut microbiome composition in 5×FAD mice. **(A–D)** *α* diversity analysis of the microbiome was performed by measuring Chao1, ACE, Shannon, and Simpson indices. **(E)** β diversity analysis was performed using PCoA. **(F,G)** Relative abundance percentages of gut microbes at the phylum and genus levels. **(H)** Clustering heatmap at the genus level. **(I,J)** Relative abundance of *Akkermansia* and *Muribaculum* at the genus level. **(K)** Phylogenetic tree generated by Linear Discriminant Analysis Effect Size (LEfSe) analysis. **(L,M)** mRNA expression of *ZO-1* and *Occludin* in mouse colon tissue. **(N–P)** Immunoblot analysis of occludin and ZO-1 in the colon. **(Q,R)** Concentrations of acetate and butyrate in mouse brain tissue. ^*^p < 0.05, ^**^p < 0.01, ^***^p < 0.001 compared to the Veh group. One-way ANOVA followed by Dunnett’s test (mean ± SEM, *n* = 3–5).

To evaluate the impact of MCT on the gut microbiota of 5×FAD mice, fecal samples were collected and analyzed using 16S rRNA sequencing. A total of 1,921,939 valid reads were generated from the four groups, with an average of 96,097 ± 3,409 valid reads for each sample. No significant differences were found in alpha diversity indices, including Chao1, ACE, Shannon, and Simpson, across the groups (Figures 4A–D). To assess whether MCT intervention altered the gut microbial composition, a beta diversity analysis was performed. Principal coordinates analysis (PCoA) revealed distinct clustering of samples between the groups (Figure 4E). Subsequently, the microbial composition was analyzed at both the phylum and genus levels. At the phylum level, a decrease in the abundance of *Firmicutes* and an increase in the abundance of *Verrucomicrobiota* were observed in the RBDCO and MCT groups compared to the vehicle-treated 5×FAD mice. At the genus level, the abundance of *Akkermansia* was significantly higher in both the RBDCO and MCT groups compared to the vehicle-treated 5×FAD mice (Figures 4F,G). The genus-level clustering heatmap revealed a significant alteration in the intestinal flora of 5×FAD mice compared to that of control mice (Figure 4H; Supplementary Figure 2). Specifically, 5×FAD mice exhibited a relatively lower abundance of *Muribaculum* and *Akkermansia*, along with higher relative abundances of *Desulfovibrio*, *Staphylococcus*, and *Helicobacter*. In contrast, MCT intervention restored the abundance of these genera (Figures 4H–J). To further characterize the dominant gut microbiota across groups, Linear Discriminant Analysis Effect Size (LEfSe) was employed, with an LDA threshold of 4, leading to the identification of 36 distinctive bacterial groups (Figure 4K; Supplementary Figure 3). In the MCT group, *Akkermansia* emerged as the dominant genus (Supplementary Figure 3), which has been shown to reduce inflammatory cytokines (25). Conversely, in the Veh group, *Bacillaceae* was the dominant genus (Supplementary Figure 3), a family known to promote inflammation (26).

The intestinal mucosal barrier plays a crucial role in protecting against the penetration of harmful substances into the blood and tissues. Tight junction proteins, such as Zonula Occludens-1 (ZO-1) and Occludin, are essential components of the intestinal barrier (27). RT-qPCR analysis of the mRNA expression levels of *ZO-1* and *Occludin* in colon tissues of mice showed that their expression was significantly decreased in the vehicle-treated 5×FAD mice compared to the control group. In contrast, MCT intervention restored the expression of these proteins to normal levels (Figures 4L,M). Notably, western blot quantification further corroborated the effects of MCT on intestinal permeability, demonstrated by significantly upregulated occludin expression and a moderate elevation in ZO-1 expression (Figures 4N–P). Reports have demonstrated that beneficial microbiota-derived short-chain fatty acids (SCFAs) can penetrate the BBB, inhibit the activation of microglia and astrocytes, prevent Aβ aggregation, reduce neuroinflammation, and alleviate the progression of AD (28). GC–MS analysis of SCFA levels in the brain tissues of mice revealed significantly higher concentrations of acetate and butyrate in the MCT group compared to the Veh group (Figures 4Q,R). These findings suggest that MCT intervention maintains intestinal flora homeostasis and restores intestinal permeability in 5×FAD mice, thereby contributing to memory improvement.

### 3.5 MCT protected primary cortical neurons against Aβ25-35-induced apoptosis and promoted neurite regeneration

**Figure 5:**
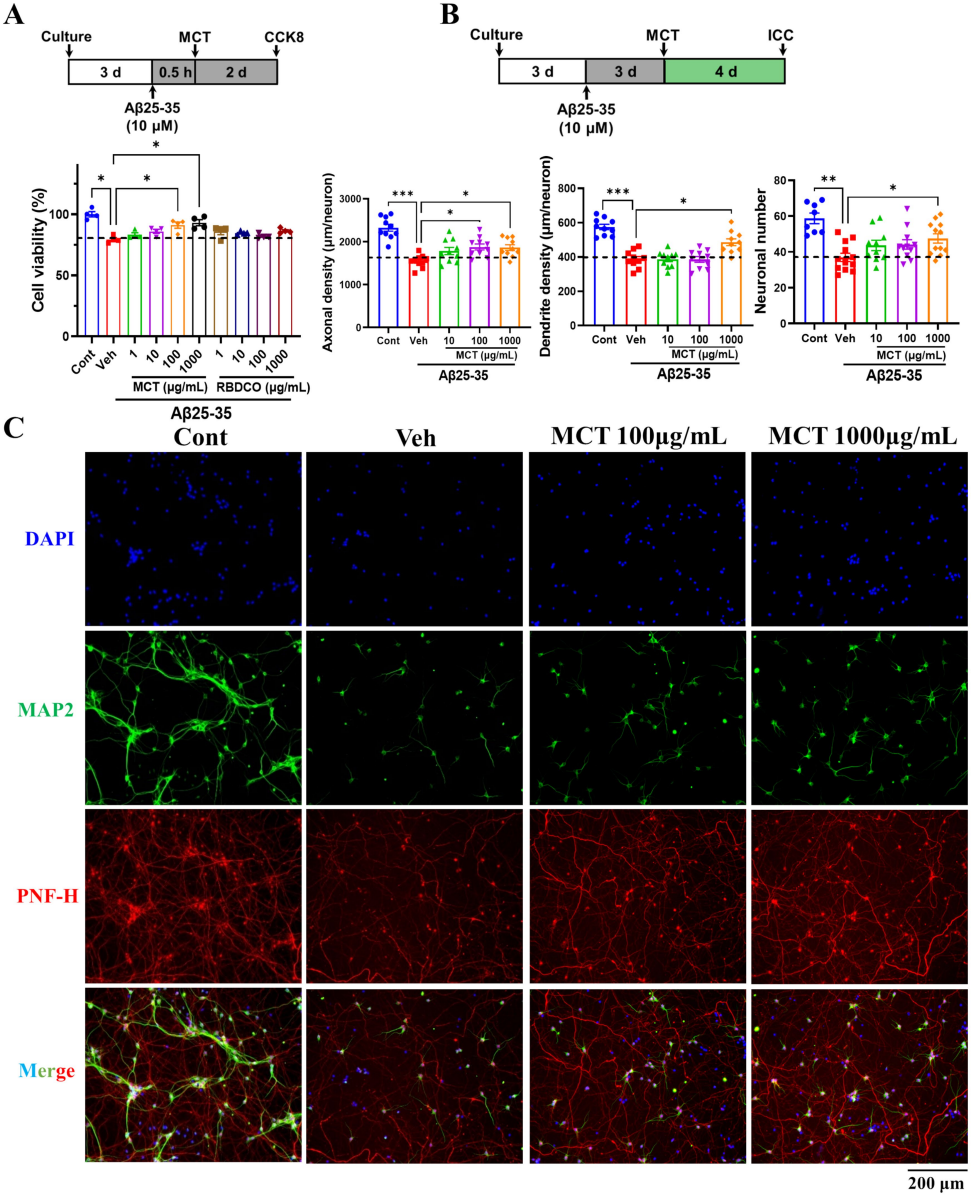
MCT alleviates Aβ25-35-induced primary neuronal injury and promotes neuronal process regeneration. **(A)** Aβ25-35 treatment for 0.5 h, MCT treatment for 2 days, then cell viability determined by CCK8. Data were normalized to the control group (mean ± SEM, *n* = 4). **(B)** Statistical results of axon density, dendrite density, and neuronal count after a 4-day treatment with MCT following 3 days of Aβ25-35 stimulation (mean ± SEM, *n* = 10–12). **(C)** Representative fluorescence images of PNF-H-positive and MAP2-positive neurons. ^*^p < 0.05, ^**^p < 0.01, ^***^p < 0.001 compared to the Veh group. One-way ANOVA followed by Dunnett’s test (mean ± SEM, *n* = 4–12).

Medium-chain fatty acids (MCFAs) are absorbed and metabolized into ketone bodies, which have a pronounced effect on mitochondrial function and neuronal survival (29). To examine the direct neuroprotective effects of MCT, an Aβ25-35-induced primary cortical neuron injury model was established, as previously described (30). Compared to the Cont group, neuronal viability was markedly reduced in the Veh group (81%). In contrast, MCT treatment significantly reversed Aβ25-35-induced neuronal death, with viability reaching 91.1% (100 μg/mL) and 93% (1,000 μg/mL) (Figure 5A).

Axons and dendrites are essential components of neural circuits and networks, playing a critical role in memory storage and retrieval (31, 32). The protective and regenerative effects of MCT on Aβ25-35-induced neurite atrophy were investigated through immunocytochemical staining. Cortical neurons were treated with 10 μM Aβ25-35 for 3 days, followed by a four-day co-treatment with MCT at concentrations of 100 and 1,000 μg/mL. The neurons were then fixed and immunostained for pNF-H and MAP2. Compared to the Cont group, axon and dendrite density in the Veh group exhibited a notable decline following Aβ25-35 treatment. Whereas, neurite density in the MCT-treated groups (100 and 1,000 μg/mL) exhibited a marked increase (Figures 5B,C). These results suggest that MCT exerts direct effects on neuronal protection and neurite regeneration.

**Figure 6:**
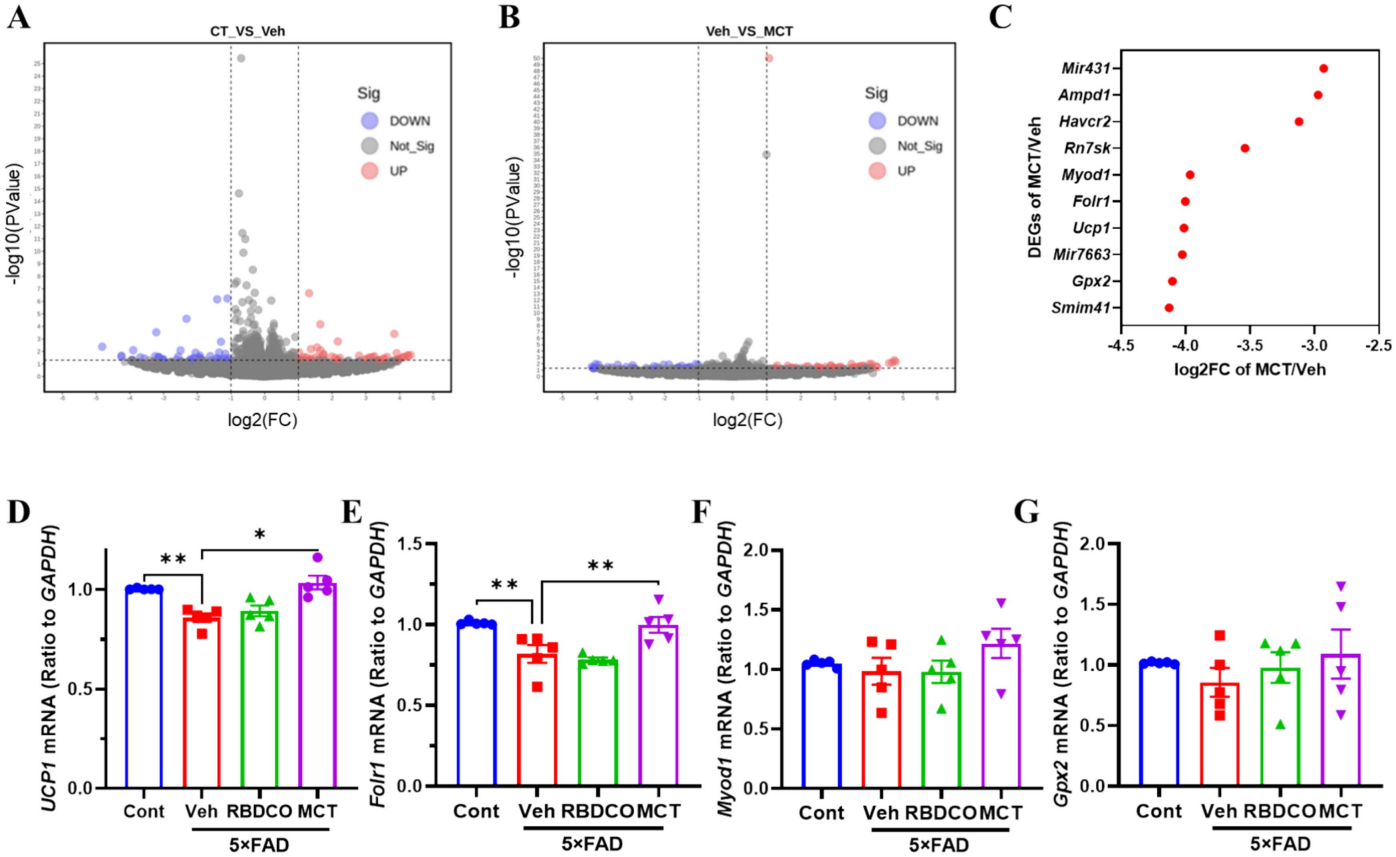
DEGs analysis and RT-qPCR validation of target gene expression. **(A)** CT vs. Veh. **(B)** Veh vs. MCT. The x-axis represents the fold change in gene expression (log2), and the y-axis represents the statistical significance of gene expression differences (−log10 *p*-value). Red points represent upregulated genes, blue points represent downregulated genes, and gray points represent genes with no significant change. **(C)** Top 10 significantly upregulated DEGs. **(D–G)** RT-qPCR validation of differentially expressed genes (*Gpx2*, *Ucp1*, *Folr1*, *Myod1*). ^*^p < 0.05, ^**^p < 0.01, ^***^p < 0.001 compared to the Veh group. One-way ANOVA followed by Dunnett’s test (mean ± SEM, *n* = 5).

A transcriptomic analysis was performed to gain insights into the potential mechanisms underlying neuronal protection by MCT in primary cultured cortical neurons. The quality of the sequencing data is presented in Supplementary Table 1, demonstrating high-quality results with a Q20 value exceeding 97% and a Q30 value exceeding 97%. A total of 93 differentially expressed genes (DEGs) were identified between the Cont and Veh groups, with 44 genes showing increased expression and 49 genes showing decreased expression (Figure 6A). Additionally, 71 DEGs were identified between the Veh and MCT groups, with 42 genes upregulated and 29 genes downregulated (Figure 6B). The top 10 most significantly regulated genes were shown in Figure 6C. Four neuron-related genes were selected for qRT-PCR validation in the brains of mice. The results revealed that uncoupling protein 1 (*UCP1*) and folate receptor 1 (*Folr1*) mRNA were dramatically decreased in vehicle-treated 5×FAD mice compared to the control group, whereas MCT intervention rescued their expression (Figures 6D,E). In contrast, no significant differences were observed for *Myod1* and *Gpx2* mRNA between the groups (Figures 6F,G). *UCP1* is involved in the regulation of neuronal apoptosis and serves as a potential therapeutic target for treating neurodegenerative diseases (33). Additionally, the increased expression of *Folr1* facilitates enhanced folate uptake into cells, and folate-mediated axon regeneration depends on the induction of *Folr1* (34). These findings suggest that both *Ucp1* and *Folr1* may serve as potential targets for the neuroprotective effects of MCT.

### 3.6 Chemical composition analysis of coconut oil derived-VCO, RBDCO, and MCT

The fatty acid composition of VCO, RBDCO and MCT was analyzed by GC–MS (Table 2). The results show that the fatty acid compositions of VCO and RBDCO are similar, with lauric acid (C12:0) being the most abundant, comprising more than 50.0%, followed by myristic acid (C14:0, ~16.0%), caprylic acid (C8:0, ~10.0%), and decanoic acid (C10, ~7.50%). In contrast, caprylic acid (C8:0) and decanoic acid (C10:0) are the two predominant fatty acids in MCT, accounting for 63.66 and 34.25%, respectively.

**Table 2:** Fatty acid composition of VCO, RBDCO, and MCT.

Fatty acids (%)	VCO	RBDCO	MCT
Caproic acid (C6:0)	1.27	1.28	1.6
Caprylic acid (C8:0)	10.06	10.57	63.66
Nonanoic acid (C9:0)	0.01	0.01	0.05
Capric acid (C10:0)	7.57	7.60	34.25
Lauric acid (C12:0)	52.33	51.96	1.69
Tridecanoic acid (C13:0)	0.03	0.03	
Myristic acid (C14:0)	16.62	16.80	0.11
Pentadecanoic acid (C15:0)	0.01	0.01	
Palmitoleic acid (C16:1, n9c)	0.04	0.04	
Palmitic acid (C16:0)	6.51	6.43	0.13
Linoleic acid (C18:2, n6c)	0.61	0.93	0.01
Oleic acid (C18:1, cis-9)	3.52	3.65	0.04
Oleic acid (C18:1, trans-9)	0.06	0.12	
Stearic acid (C18:0)	2.50	1.78	0.07
Arachidic acid (C20:0)	0.05	0.03	
Lignoceric acid (C24:0)	0.06	0.03	

LC–MS/MS was performed to determine the polyphenolic acid contents. The results revealed that VCO had the highest total phenolic acid content (1608.6 μg/kg), followed by RBDCO (567.6 μg/kg) and MCT (431.1 μg/kg). The individual polyphenols identified were cinnamic acid, *p*-coumaric acid, hydroxybenzoic acid, protocatechuic acid, erucic acid, and vanillic acid, with cinnamic acid being the most abundant (Supplementary Table 2).

Tocopherols (vitamin E) are lipid-soluble natural antioxidants found in most vegetable oils. Among various vegetable oils, coconut oil contains relatively low tocopherol levels, as its low unsaturated fatty acid content makes it less prone to autooxidation. Supplementary Table 3 shows that VCO and RBDCO contained similar amounts of *α*-tocopherol and *γ*-tocopherol, while MCT had relatively low tocopherol levels.

## 4 Discussion

AD is the most common form of dementia, with a highly intricate etiology that impacts a considerable portion of the aged individuals (35) Dietary interventions, being low-risk and cost-effective, represent promising strategies for mitigating AD (36, 37). Current evidence suggests that coconut oil may offer a viable intervention with broad potential (38, 39). However, it remains unclear whether coconut oil exerts neuroprotective effects in AD, and if so, which components are effective and what the underlying mechanisms might be. The present study demonstrates that MCT and RBDCO, but not VCO, improve learning and memory abilities in 5×FAD mice, with MCT showing superior effects over RBDCO. MCT reduced Aβ accumulation, glial cell hyperactivation, hippocampal neuronal damage, and maintained gut microbiota homeostasis. Furthermore, MCT restored gut barrier integrity, accelerated the release of anti-inflammatory factors and brain short-chain fatty acids, promoted neuronal protection and neurite regeneration, and ultimately alleviated memory deficits in 5×FAD mice.

The 5×FAD mouse model is one of the most widely used models for AD research. These mice begin to accumulate Aβ deposits at 2 months of age and develop memory deficits by 6 months, along with severe amyloid plaque, tau accumulation, and synaptic loss (40). In this study, we used 5×FAD mice aged 6–8 months. As expected, these mice exhibited deficits in learning and spatial memory, as assessed by the ORT, OLT, and Morris Water Maze test. Aβ is a key pathological marker of AD and plays a pivotal role in its progression. Excessive Aβ accumulation promotes the formation of neurofibrillary tangles and plaques, leading to neuronal dysfunction and death (41). A recent study indicates that the MCFAs enhance the degradation of Aβ by increasing the secretion and enzymatic activity of insulin-degrading enzyme (IDE) (42). Our study further confirmed that MCT supplementation, but not RBDCO intervention, resulted in a reduction of Aβ plaques in the brains of 5×FAD mice.

Mounting evidence suggests that the gut microbiota plays a pivotal role in the pathogenesis of AD (43). Dysbiosis can lead to systemic inflammation and compromise the integrity of the intestinal epithelial barrier, further exacerbating AD pathology and cognitive decline (44, 45). The gut microbiota is a key player that interacts with the host through the production of diverse metabolites, including SCFAs such as acetate, propionate, and butyrate, as well as metabolites derived from linoleic acid and tryptophan (46). These metabolites play crucial roles in regulating immune responses, cell cycle, and neuronal signaling, all of which are highly relevant to the progression of AD (47). To assess the effects of MCT intervention on the gut microbiota, we conducted 16S rRNA gene sequencing. The results revealed that MCT intervention led to significant changes in the composition of the gut microbiota. At the phylum level, the MCT-treated mice exhibited a notable increase in the abundance of *Verrucomicrobiota*, reaching 35.1%, which represented a 26.3% increase compared to the vehicle-treated 5×FAD mice. Previous studies have shown that certain members of *Verrucomicrobiota*, including *Akkermansia muciniphila*, are capable of producing SCFAs (48). Furthermore, LEfSe analysis identified 20 bacterial species that responded to MCT treatment. Among these, *Akkermansia muciniphila* is one of the most abundant bacteria within the *Verrucomicrobiota* genus, known for its diverse beneficial properties (49). A reduction in the abundance of *Akkermansia muciniphila* has been observed in AD patients (50). Additionally, studies have shown that oral administration of *Akkermansia muciniphila* to mice for 2 weeks significantly increased the expression of *Occludin* and *Tjp-1*, strengthening the intestinal barrier and reducing LPS-induced toxicity (51). In the present study, the abundance of *Akkermansia muciniphila* was lower in the Veh group compared to the Cont group. However, MCT treatment significantly restored its abundance, suggesting that MCT helps to rebalance gut microbiota and restore gut homeostasis. Moreover, GC–MS analysis and qRT-PCR results revealed that MCT intervention significantly increased acetate and butyrate levels in the brain and elevated the mRNA expression of *ZO-1* and *Occludin* in the colon compared to the Veh group. Western blot analysis further substantiated the effects of MCT intervention, with a significant upregulation of occludin protein and an upward trend in the protein expression of ZO-1. These findings suggest that MCT restores the intestinal barrier integrity and boosts SCFAs by modulating the gut microbiota, thereby alleviating AD symptoms.

Microglia and astrocytes, the primary resident immune cells in the central nervous system (CNS), play crucial roles in modulating neuroinflammatory responses (52). Persistent neuroinflammation can result in synaptic dysfunction and exacerbate neuronal death in AD, thereby accelerating cognitive decline (53). Therefore, inhibiting the overactivation of glial cells and reducing the production of pro-inflammatory factors are essential strategies for slowing the progression of AD and mitigating neuronal damage. Our study demonstrated for the first time that MCT intervention reduced the number of Iba1^+^ microglia and GFAP^+^ astrocytes, suggesting that MCT may alleviate neuroinflammation by reducing the hyperactivation of glial cells. The intestinal barrier integrity plays a crucial role in defending against external toxins, thereby promoting higher circulating levels of anti-inflammatory cytokines produced by immune cells in response to microbiota-derived metabolites.

Previous reports have shown that MCT supplementation attenuates Aβ deposition and neuronal apoptosis by enhancing brain glucose metabolism in APP/PS1 mice (54). In the present study, we confirmed that MCT intervention significantly preserved NeuN^+^ neurons in the hippocampus compared to vehicle-treated 5×FAD mice. The intrinsic cell apoptosis pathway is regulated by the *Bcl-2* gene family, which includes two major subclasses of proteins *Bcl-2* (anti-apoptotic) and *Bax* (pro-apoptotic) (55). qRT-PCR results revealed that MCT treatment increased *Bcl-2* mRNA expression and decreased *Bax* mRNA expression compared to vehicle-treated 5×FAD mice, suggesting that MCT intervention may protect neurons by reducing hippocampal neuronal apoptosis in 5×FAD mice. Accumulating evidence implicates MAP2 and PSD-95 as critical regulators of neurostructural plasticity, particularly in axonal elongation dynamics (56). Our biochemical analyses revealed that MCT intervention elicited a significant elevation in MAP2 expression, accompanied by an increasing tendency in PSD-95 levels. These findings collectively imply that MCT-mediated cytoskeletal stabilization and synaptic remodeling may establish a permissive microenvironment conducive to neural repair processes. Nevertheless, it should be emphasized that while enhanced expression of these plasticity-related biomarkers provides circumstantial evidence, it does not constitute direct demonstration of axonal regeneration *in vivo*. To address this mechanistic gap, subsequent investigations will employ combinatorial strategies incorporating: (1) growth-associated protein-43 (GAP-43) immunolabeling for monitoring nascent axonal sprouts; (2) anterograde neurotracing with biotinylated dextran amine to map axonal trajectory remodeling; and (3) transmission electron microscope for ultrastructural characterization of synaptic active zones.

The regeneration of axons and dendrites is crucial for memory formation, consolidation, and retrieval. Our previous findings demonstrated a positive correlation between the promotion of axonal elongation and memory improvement in AD (16). Compounds that stimulate axonal elongation *in vitro* have been shown to significantly enhance cognitive and spatial memory in normal mice. Moreover, clinical studies indicate that these compounds can significantly improve memory in healthy individuals (57). Aβ-induced neuronal death disrupts the neural network, ultimately leading to memory deficits. Thus, we established an Aβ25-35-induced primary cortical neuronal injury and neurite atrophy model to examine the effects of MCT on cell survival and neurite regeneration. MCT intervention effectively reversed Aβ25-35-induced neuronal death, as well as a decrease of MAP2-positive dendrites and pNF-H-positive axons. Caprylic acid and capric acid, the predominant components in MCT, play important roles in neuronal function. Caprylic acid promotes neurite outgrowth in PC12 cells via the activation of p38 MAPK and ERK pathways (58). This study provides the first evidence that MCT directly protects cortical neurons against Aβ-induced neuronal apoptosis and neurite atrophy.

RNA sequencing was performed to explore potential targets of MCT in neuronal protection. A total of 71 differentially expressed genes were obtained between the Veh and MCT groups, with the top four neuron-related genes selected for qRT-PCR validation. The results revealed that *Ucp1* and *Folr1* mRNA levels were significantly upregulated post MCT treatment compared to vehicle-treated 5×FAD mice. Evidence has shown that increasing *UCP1* expression can inhibit apoptosis and potentially treat neurological disorders. Additionally, deletion of *UCP1* in Tg2576 mice exacerbates AD-related pathologies (59). *Folr1* plays a crucial role in axonal growth during spinal cord development, and its knockdown in embryonic spinal cord explants leads to axon constriction and a reduction of axonal β3-tubulin protein (60). A notable methodological limitation in our investigation pertains to the exclusive focus on RNA-seq profiling in central nervous system (CNS)-derived neuronal cultures, which precludes extrapolation to enteric nervous system (ENS) components. While both CNS and ENS share fundamental neural signaling mechanisms, emerging evidence reveals profound functional and transcriptional differences between these distinct neural populations, particularly in neurotransmitter repertoire and microenvironmental responsiveness. Thus, our study failed to differentiate between pan-neuronal regulatory patterns and region-specific adaptations in MCT-mediated neuromodulation. Future investigations will require multi-omics approaches to achieve systems-level insights into the neuro-enteric crosstalk mechanisms underlying MCT signaling, thereby enabling more precise therapeutic targeting of gut-brain axis disorders—from neurodegenerative conditions to enteric neuropathies.

**Figure 7:**
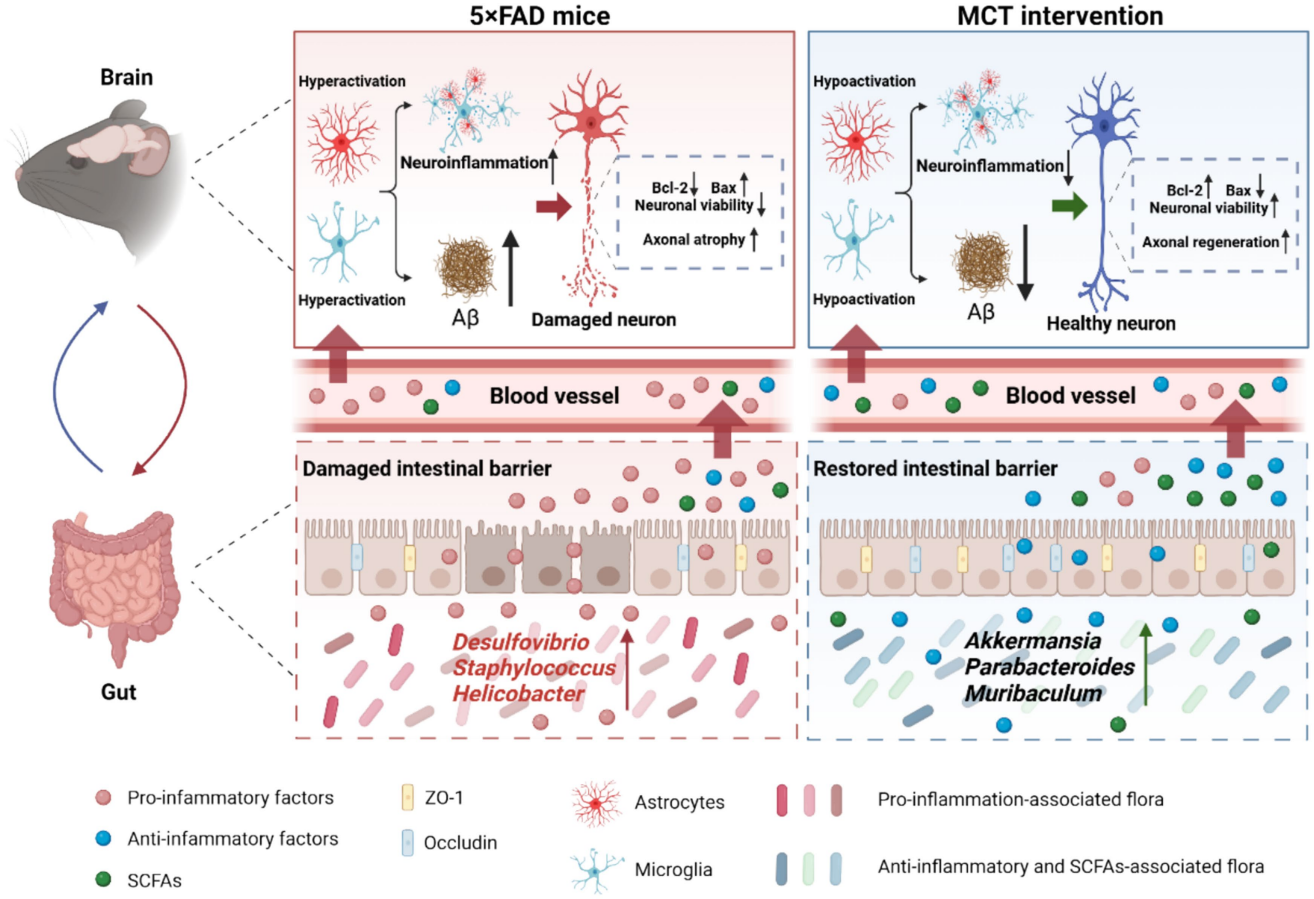
MCT ameliorated memory deficits via promoting neurite outgrowth and maintaining gut homeostasis in 5×FAD mice. MCT intervention increased the relative abundance of probiotics and restored intestinal permeability, resulting in a reduction of pro-inflammatory cytokines (IL-6) and an increase in anti-inflammatory cytokines (IL-4 and IL-10) as well as SCFAs (acetic and butyric acids). These changes helped to mitigate neuroinflammation and Aβ deposition. Additionally, MCT reduced neuronal apoptosis and promoted axonal regeneration, contributing to cognitive improvements.

In conclusion, MCT intervention modulated the intestinal microbiota composition in 5×FAD mice by increasing the relative abundance of *Akkermansia muciniphila*, thereby restoring reduced brain levels of SCFAs. Additionally, MCT improved intestinal permeability by upregulating the expression of tight junction proteins ZO-1 and Occludin, helping maintain a balanced systemic immune response. The beneficial systemic cytokines further inhibited neuroinflammation by suppressing hyper-activated microglia and astrocytes, which may contribute to Aβ plaque reduction and enhanced neuroprotection. Moreover, MCT directly inhibited Aβ25-35-induced neuronal death and neurite atrophy, possibly through the upregulation of *Ucp1* and *Folr1* expression (Figure 7). Our findings suggest that MCT could serve as a potential dietary therapy for preventing or treating AD.

## Data Availability

The datasets presented in this study can be found in online repositories. The names of the repository/repositories and accession number(s) can be found in the article/Supplementary material.
