# Supplementary material for: Coconut oil derived medium-chain triglycerides ameliorated memory deficits via promoting neurite outgrowth and maintaining gut homeostasis in 5×FAD mice
Source: Front Nutr. 2025 Jun 2;12:1585640. doi: 10.3389/fnut.2025.1585640 (PMC12171442; doi:10.3389/fnut.2025.1585640)

Supplementary Materials

Coconut oil derived medium-chain triglycerides ameliorated memory deficits via promoting neurite outgrowth and maintaining gut homeostasis in 5×FAD mice

Ruiye Chen[a,#], Rui Li[a,#], Jiahui Jiang[a], Longjian Zhou[a], Shuai Zhao[a], Yi Zhang[a], Qiuyu Xia[a], Zhiyou Yang[a,*]

[a]College of Food Science and Technology, Shenzhen Institute of Guangdong Ocean University, Guangdong Provincial Key Laboratory of Aquatic Product Processing and Safety, Guangdong Province Engineering Laboratory for Marine Biological Products, Zhanjiang Municipal Key laboratory of Marine Drugs and Nutrition for Brain Health, Zhanjiang 524088, China.

*Corresponding author:

Zhiyou Yang, PhD, College of Food Science and Technology, Guangdong Ocean University, Zhanjiang 524088, China. Tel./fax: +86-0759-2396046. E-mail address: yang_zhiyou@sina.com

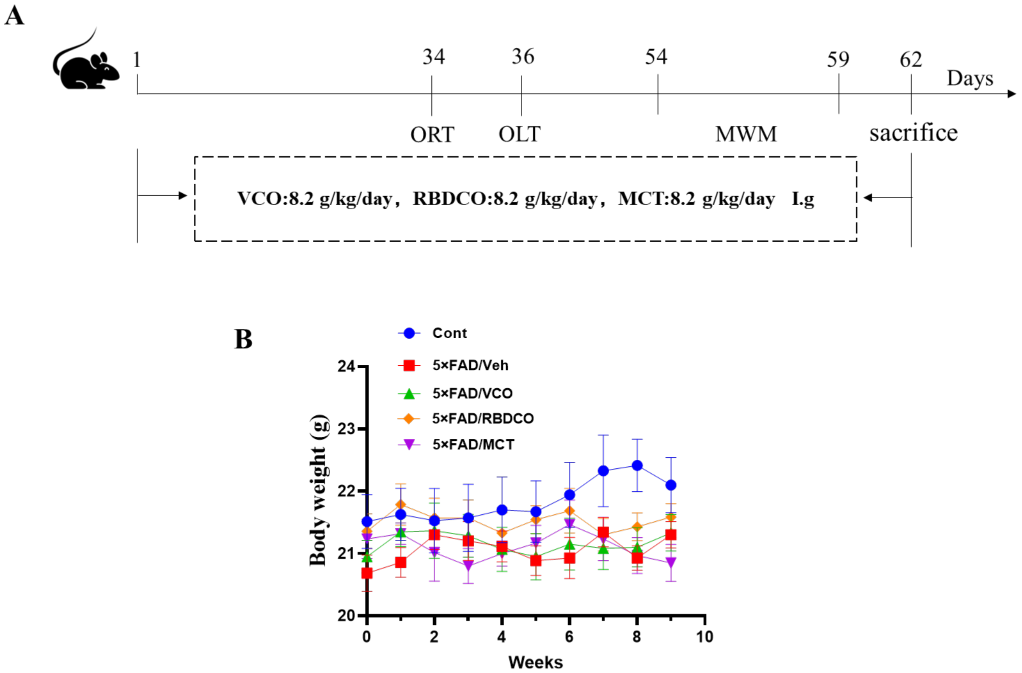

Supplementary Fig. 1. MCT and RBDCO ameliorated memory deficits in 5×FAD mice. (A) The experimental time schedule. VCO, RBDCO, and MCT (8.2 g/kg/day) were orally administered to 5×FAD mice (female, 6-8 months old, n = 6-7). (B) Body weight changes in mice during VCO, RBDCO and MCT treatments.

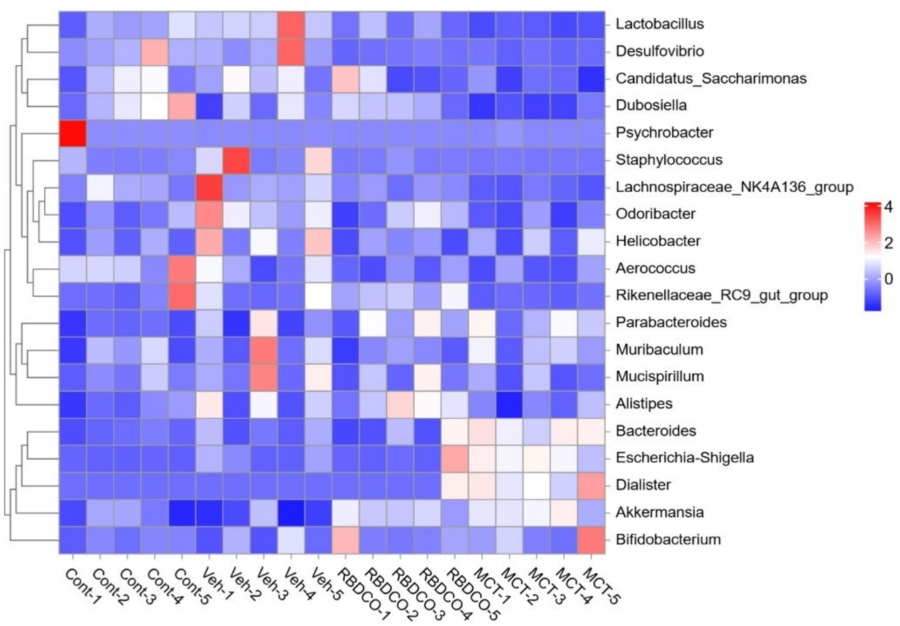

Supplementary Fig. 2. The clustering heatmap of genus-level species.

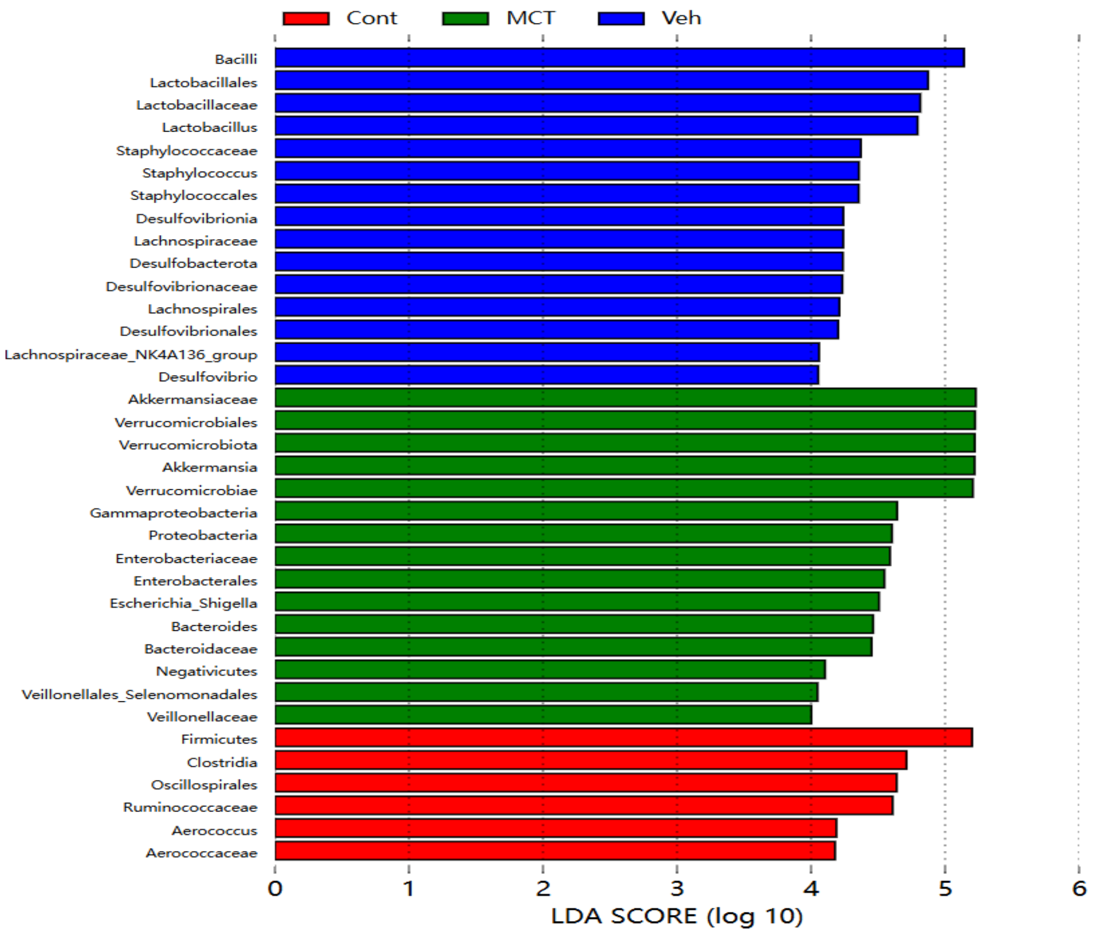

Supplementary Fig. 3. LEfSe analysis of MCT-altered biomarker taxa. LDA scores of microbial clades that differ in relative abundance among Cont (red), Veh (blue), and MCT (green) groups (with LDA scores > 4 and p-values < 0.05, determined using the Kruskal-Wallis test).

Supplementary Table 1. The quality of RNA-seq data

	Before reads	After reads	After reads%	Q20%	Q30%
Cont-1	49113190	48796124	99.35	0.980331	0.980331
Cont-2	43476538	43205212	99.38	0.980089	0.980089
Cont-3	50924616	50579256	99.32	0.978691	0.978691
Veh-1	47177932	46891544	99.39	0.979463	0.979463
Veh-2	43884372	43616554	99.39	0.979399	0.979399
Veh-3	45732968	45449432	99.38	0.979421	0.979421
MCT-1	42700202	42414828	99.33	0.978614	0.978614
MCT-2	47779440	47455548	99.32	0.977744	0.977744
MCT-3	47852494	47526948	99.32	0.977863	0.977863

Supplementary Table 2. Phenolic profiles of VCO, RBDCO, and MCT

**Polyphenols (μg/kg)**	**VCO**	**RBDCO**	**MCT**
Cinnamic acid	1440.0	429.0	247.0
p-Coumaric acid	63.9	60.8	97.8
Hydroxybenzoic acid	36.1	N/A	35.9
Protocatechuic acid	32.0	30.1	30.5
Erucic acid	21.8	N/A	N/A
Vanillic acid	14.8	47.7	19.9
Total	1608.6	567.6	431.1

Supplementary Table 3. Tocopherol composition and content of VCO, RBDCO, and MCT

Samples	Content (mg/kg)		
	α-Tocopherol	γ-Tocopherol	Total
VCO	5.54	12.54	18.08
RBDCO	5.07	13.26	18.33
MCT	2.03	8.06	10.09

Original immunoblots in Fig 3E

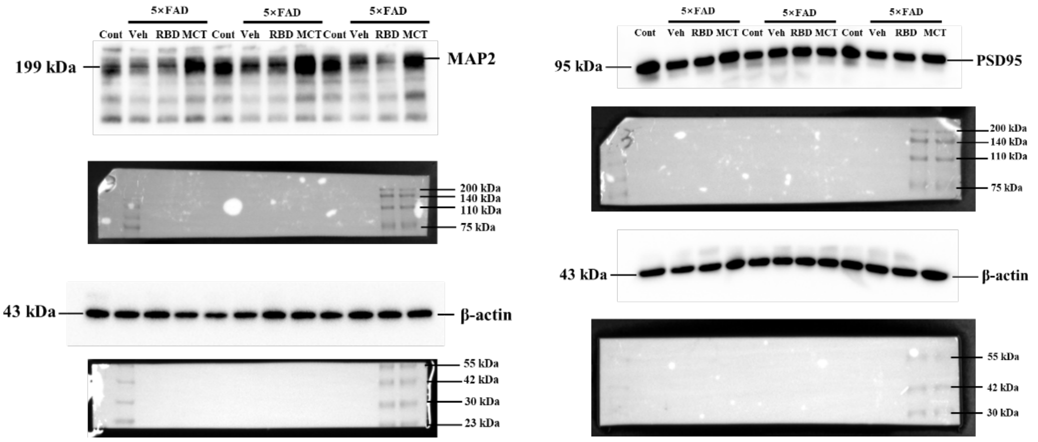

Original immunoblots in Fig 4N

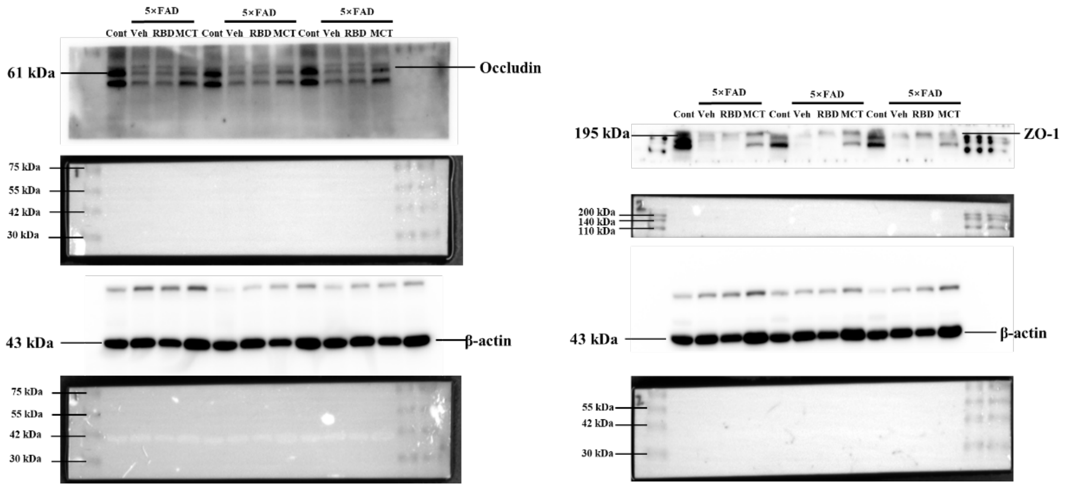

Supplement: Supplementary file 1 [file Image_1.pdf]
