# Supplementary material for: Coconut oil derived medium-chain triglycerides ameliorated memory deficits via promoting neurite outgrowth and maintaining gut homeostasis in 5×FAD mice
Source: Front Nutr. 2025 Jun 2;12:1585640. doi: 10.3389/fnut.2025.1585640 (PMC12171442; doi:10.3389/fnut.2025.1585640)

## Slide 1
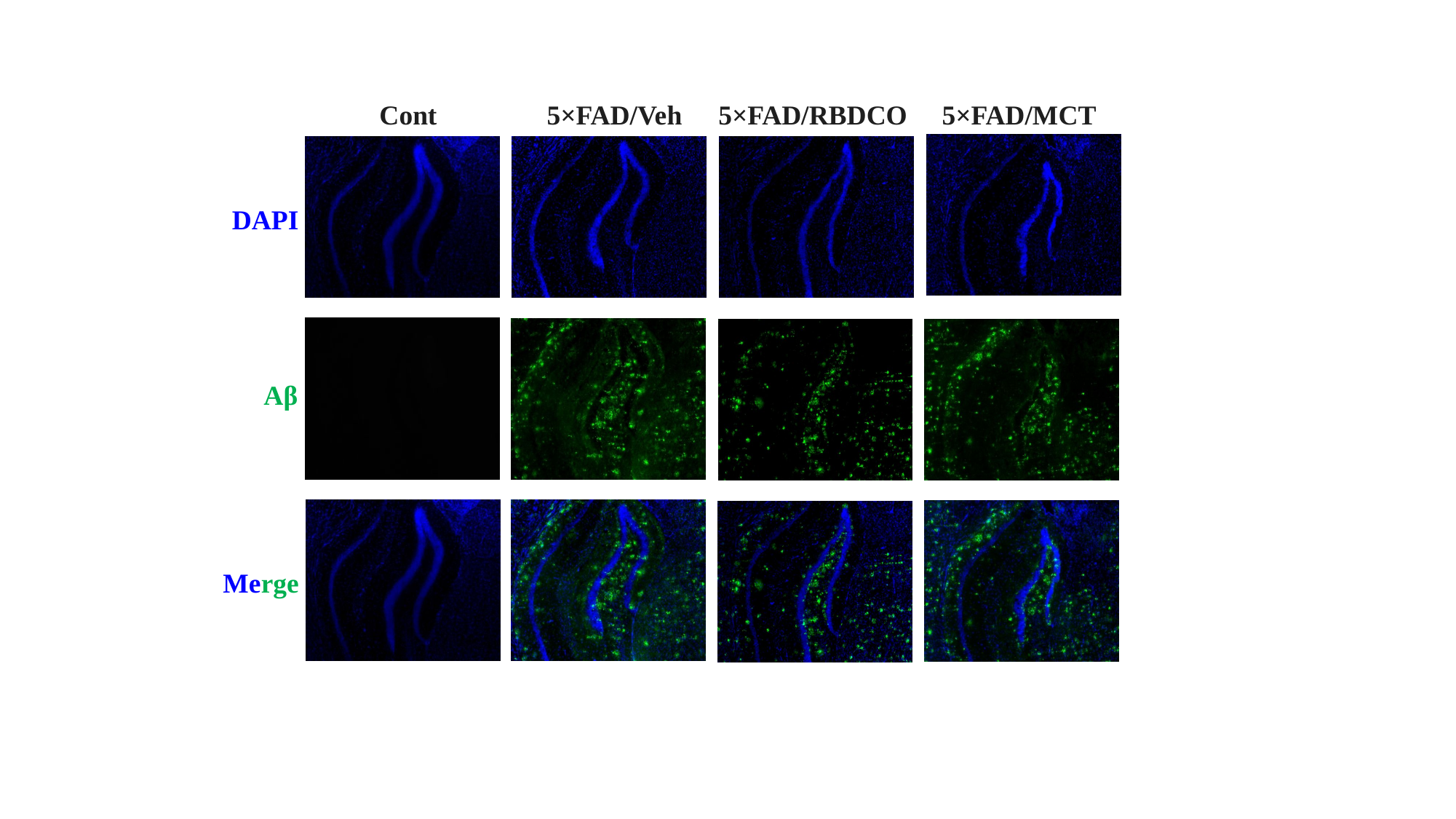

Cont
5×FAD/Veh
5×FAD/RBDCO
5×FAD/MCT
DAPI
Aβ
Merge

## Slide 2
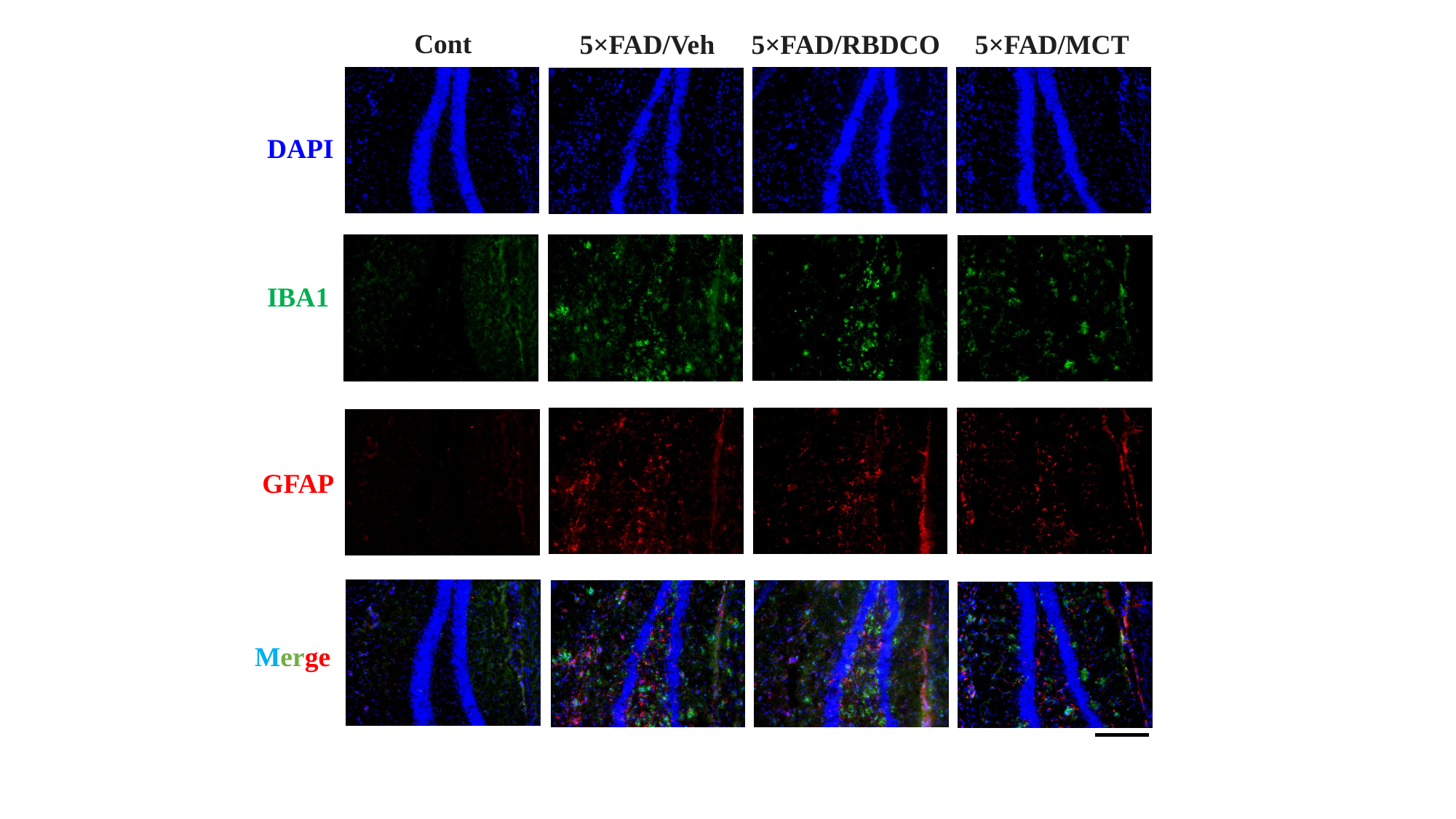

Cont
5×FAD/Veh
5×FAD/RBDCO
5×FAD/MCT
DAPI
IBA1
GFAP
Merge

## Slide 3
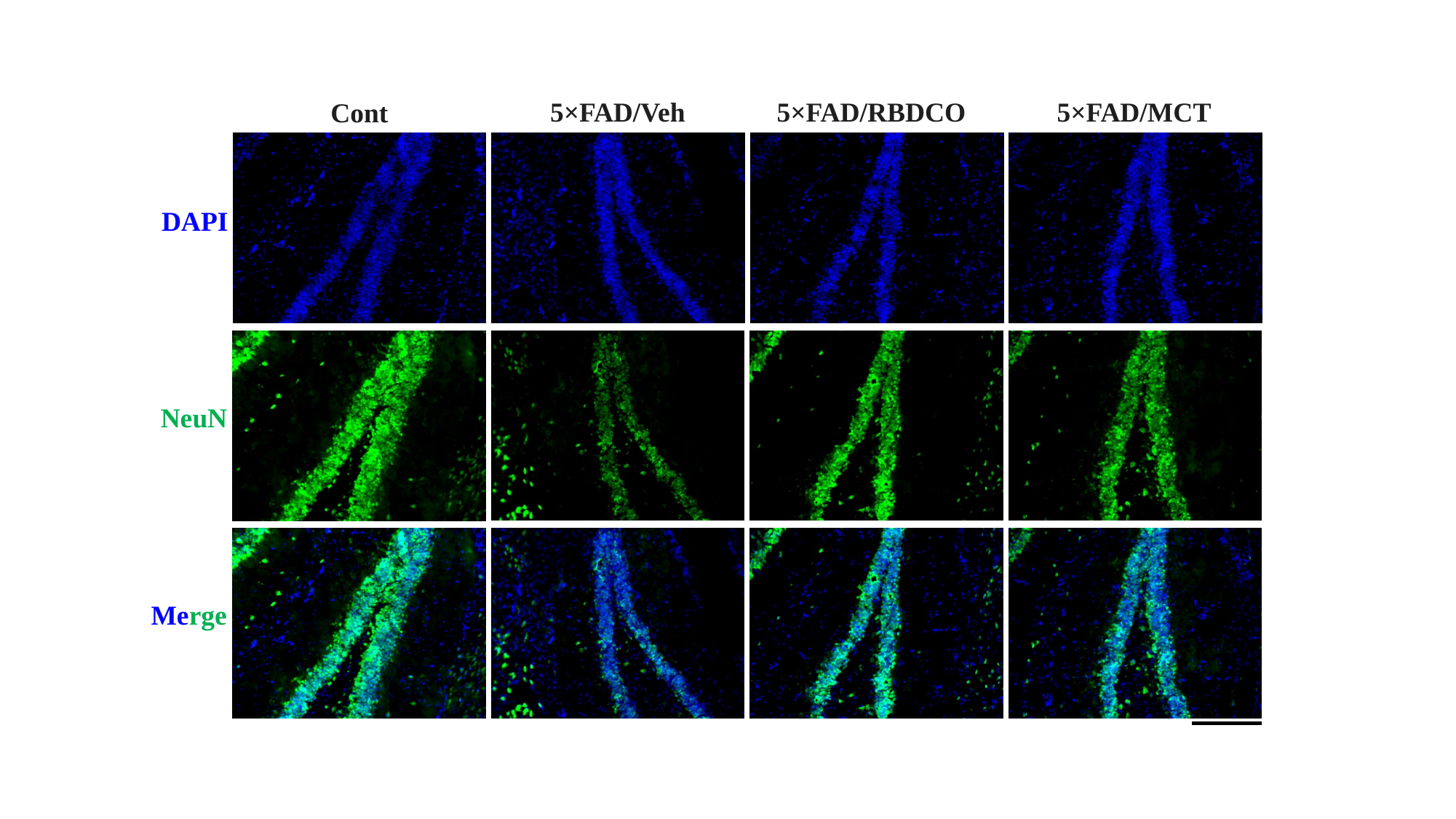

5×FAD/Veh
5×FAD/RBDCO
5×FAD/MCT
Cont
DAPI
NeuN
Merge

## Slide 4
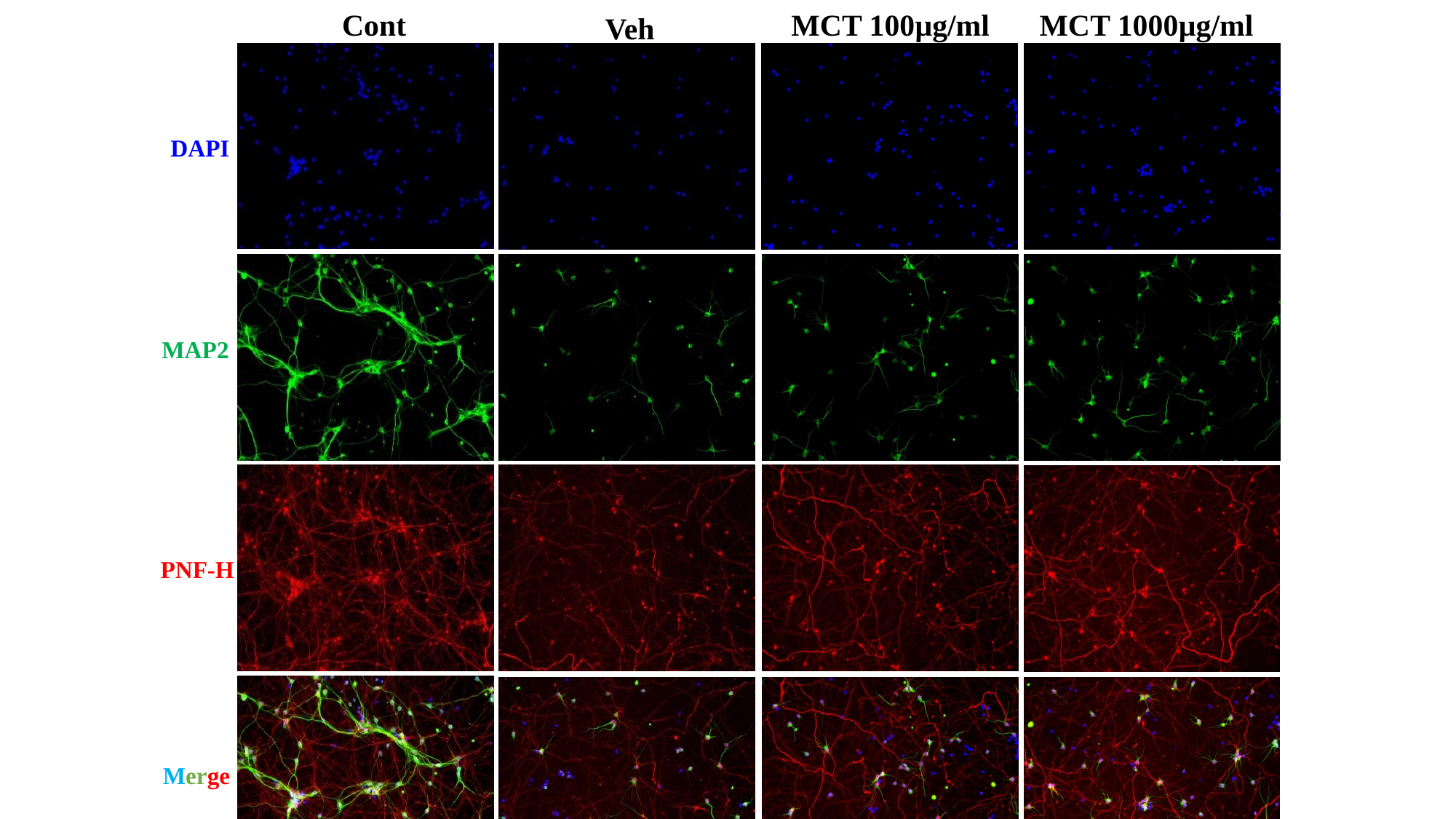

Cont
 MCT 100μg/ml
 MCT 1000μg/ml
Veh
DAPI
MAP2
PNF-H
Merge
200 μm

## Slide 5
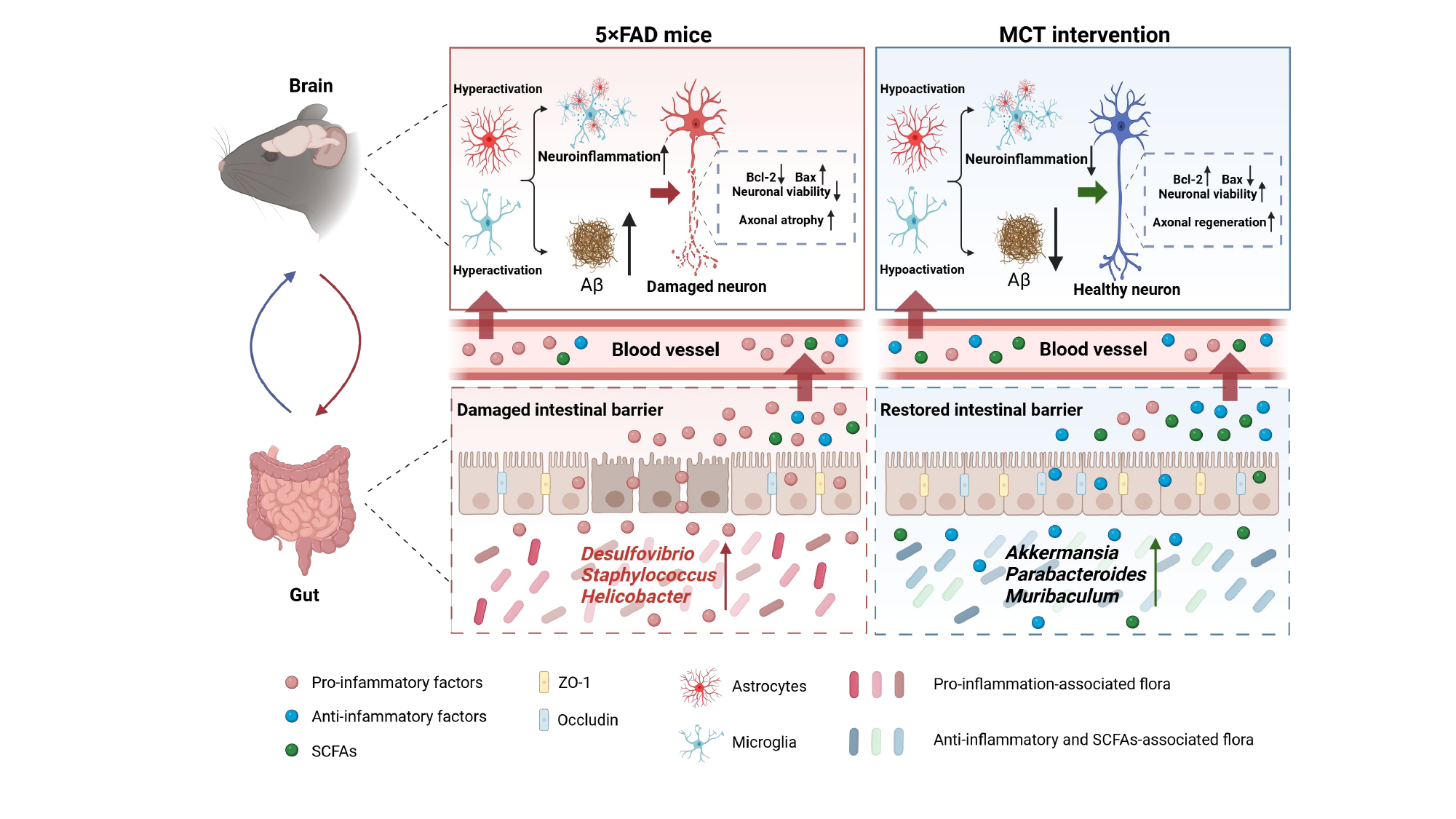

Supplement: Supplementary file 2 [file Data_Sheet_1.zip › Immunofluorescence pictures.pptx]
